# Cloning and Functional Characterization of the Polyketide Synthases Based on Genome Mining of *Preussia isomera* XL-1326

**DOI:** 10.3389/fmicb.2022.819086

**Published:** 2022-05-04

**Authors:** Qingpei Liu, Dan Zhang, Yao Xu, Shuaibiao Gao, Yifu Gong, Xianhua Cai, Ming Yao, Xiaolong Yang

**Affiliations:** The Modernization Engineering Technology Research Center of Ethnic Minority Medicine of Hubei Province, School of Pharmaceutical Sciences, South-Central Minzu University, Wuhan, China

**Keywords:** *Preussia isomera*, genome mining, fungal polyketides, polyketide synthase, heterologous expression, orsellinic acid derivatives

## Abstract

Fungal polyketides (PKs) are one of the largest families of structurally diverse bioactive natural products biosynthesized by multidomain megasynthases, in which thioesterase (TE) domains act as nonequivalent decision gates determining both the shape and the yield of the polyketide intermediate. The endophytic fungus *Preussia isomera* XL-1326 was discovered to have an excellent capacity for secreting diverse bioactive PKs, i.e., the hot enantiomers (±)-preuisolactone A with antibacterial activity, the single-spiro minimoidione B with α-glucosidase inhibition activity, and the uncommon heptaketide setosol with antifungal activity, which drive us to illustrate how the unique PKs are biosynthesized. In this study, we first reported the genome sequence information of *P. isomera*. Based on genome mining, we discovered nine transcriptionally active genes encoding polyketide synthases (PKSs), Preu1–Preu9, of which those of Preu3, Preu4, and Preu6 were cloned and functionally characterized due to possessing complete sets of synthetic and release domains. Through heterologous expression in *Saccharomyces cerevisiae*, Preu3 and Preu6 could release high yields of orsellinic acid (OA) derivatives [3-methylorsellinic acid (3-MOA) and lecanoric acid, respectively]. Correspondingly, we found that Preu3 and Preu6 were clustered into OA derivative synthase groups by phylogenetic analysis. Next, with TE domain swapping, we constructed a novel “non-native” PKS, Preu6-TE_Preu3_, which shared a very low identity with OA synthase, OrsA, from *Aspergillus nidulans* but could produce a large amount of OA. In addition, with the use of Preu6-TE_Preu3_, we synthesized methyl 3-methylorsellinate (synthetic oak moss of great economic value) from 3-MOA as the substrate, and interestingly, 3-MOA exhibited remarkable antibacterial activities, while methyl 3-methylorsellinate displayed broad-spectrum antifungal activity. Taken together, we identified two novel PKSs to biosynthesize 3-MOA and lecanoric acid, respectively, with information on such kinds of PKSs rarely reported, and constructed one novel “non-native” PKS to largely biosynthesize OA. This work is our first step to explore the biosynthesis of the PKs in *P. isomera*, and it also provides a new platform for high-level environment-friendly production of OA derivatives and the development of new antimicrobial agents.

## Introduction

Fungal polyketides (PKs) are one of the largest classes of small-molecule natural products with diverse structures and various interesting biological activities. They can typically be classified into naphthopyrones, isocoumarins, acetyl tetrahydroxynaphthalene (ATHN), anthraquinones, benzenediol lactones (BDLs), and orsellinic acid (OA) derivatives (Lackner et al., 2013; Hussain et al., 2017; Wang et al., 2020). Importantly, they also provide lead compounds and inspiration for pharmaceutical drug discovery, which is evidenced by the cholesterol-lowering agents/statins, the antibiotic griseofulvin, and the immunosuppressant mycophenolic acid (Keller, 2019). Fungal PKs are biosynthesized by multidomain megasynthases [type I iterative polyketide synthases (iPKSs)], which may be classified into three subgroups: non-reducing iPKSs (nrPKSs), partially reducing iPKSs, and highly reducing iPKSs (hrPKSs), according to the reduction degree of the PKs (Xu et al., 2013b). nrPKSs usually harbor a set of loading component: starter unit ACP transacylase (SAT) domain; chain extension components consisting of ketoacyl synthase (KS), acyl transferase (AT), product template (PT), and acyl carrier protein (ACP) domains; and processing components including methyl transferase (CMeT) and thioesterase (TE) or reductive release (R) domains (Cox, 2007; Bai et al., 2016). hrPKSs generally possess ketoreductase (KR), dehydratase (DH), and enoyl reductase (ER) domains to reduce the nascent β-ketoacyl intermediates into β-alcohol, the alkene, or alkane after each condensation process (Xu et al., 2013b).

**Figure 1:**
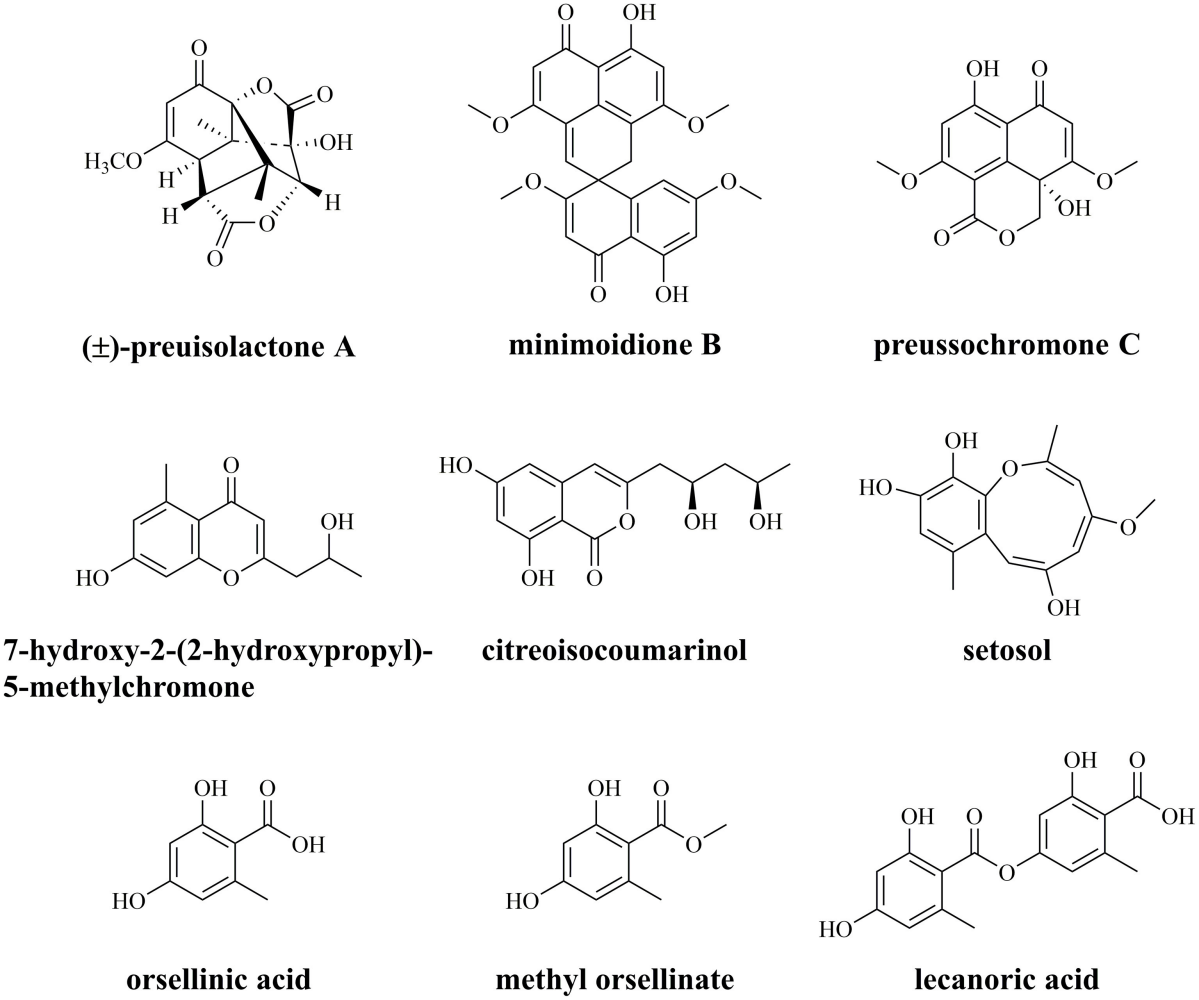
Characterized polyketides from endophytic fungus *Preussia isomera* XL-1326.

The endophytic fungus *Preussia isomera* XL-1326, isolated from the stems of *Panax notoginseng*, has an excellent capacity for secreting diverse PKs with various biological activities (Figure 1) (Xu et al., 2019; Chen et al., 2020). The most famous one is (±)-preuisolactone A with remarkable antibacterial activity, first reported as a sesquiterpenoid by our group, while suggested as a phenolic PK *via* biomimetic synthesis by other groups (Novak et al., 2019; Xu et al., 2019; Zhang et al., 2021). *P. isomera* XL-1326 could also produce several PKs with unique structures, including minimoidione B, a single-spiro compound with strong α-glucosidase inhibitory activity (Rangel-Grimaldo et al., 2017); preussochromone C, a chromone derivative with significant cytotoxicity and α-glucosidase inhibitory activity (Zhang et al., 2012; Rangel-Grimaldo et al., 2017); 7-hydroxy-2-(2-hydroxypropyl)-5-methylchromone, a chromone derivative with no detected cytotoxicity or antimicrobial activity (Xu et al., 2009; Guo et al., 2021); citreoisocoumarinol, an isocoumarin with moderate α-glucosidase inhibitory activity (Cui et al., 2016); and setosol, an uncommon heptaketide compound with antifungal activity (Okeke et al., 1995). In addition, we also identified OA and its derivatives, methyl orsellinate and lecanoric acid (LA), from *P. isomera* XL-1326 (Figure 1, unpublished data). What intrigued us most is how the unique PKs are biosynthesized, and how the specific chemical reactions are catalyzed during the biosynthesis, i.e., the (5+2)-cycloaddition in the biosynthesis of (±)-preuisolactone A, the spiro reaction in the biosynthesis of minimoidione B, and the ring expansion reaction in the biosynthesis of setosol.

In the current study, to preliminarily explore the biosynthesis of the PKs, we performed a comprehensive bioinformatic analysis of the genome of *P. isomera* XL-1326 and discovered nine transcriptionally active iPKSs, Preu1–Preu9, of which Preu3 and Preu6 were identified to biosynthesize 3-methylorsellinic acid (3-MOA) and LA through heterologous expression, respectively. With a combinatorial biosynthesis strategy, we constructed a novel “non-native” PKS, Preu6-TE_Preu3_, which could largely biosynthesize OA. In addition, we synthesized methyl 3-methylorsellinate utilizing 3-MOA and showed that 3-MOA exhibited remarkable antibacterial activity, while methyl 3-methylorsellinate displayed broad-spectrum antifungal activity. This work is our first step to explore the biosynthesis of the PKs in *P. isomera* XL-1326, and it also provides a new platform for the high-level environment-friendly production of OA derivatives and the development of new antimicrobial agents.

## Materials and Methods

### Fungal Strain and Genome Sequencing

The fungus *Preussia isomera* XL-1326 was isolated from the stems of *Panax notoginseng* collected in Wenshan, Yunnan Province, China, and was identified by analysis of the ITS region of rDNA (GenBank accession no: MK300824.1) (Xu et al., 2019; Chen et al., 2020). Fungal genomic DNA was isolated from mycelia grown on cellophane membranes covering potato dextrose agar (PDA) plates, using the cetyltrimethylammonium bromide (CTAB) method (Shao et al., 2009). The whole-genome sequencing of *P. isomera* XL-1326 was performed on PacBio Sequel platform by Novogene Co., Ltd (Beijing, China).

### Bioinformatic Methods

The whole-genome sequence of *P. isomera* XL-1326 was submitted to antiSMASH (version 5.0.0; Blin et al., 2019) for SM gene cluster characterization. The amino acid sequences encoded by the *pks* genes were deduced using FGENESH (http://linux1.softberry.com/berry.phtml) and analyzed using Pfam 27.0 (http://pfam.sanger.ac.uk/) and CD-Search (https://www.ncbi.nlm.nih.gov/Structure/cdd/wrpsb.cgi). Similarities among the deduced amino acid sequences were analyzed using BLASTP (http://blast.ncbi.nlm.nih.gov/Blast.cgi). The amino acid sequences of the PKSs are listed in the Supplementary Material.

Sequences of representative fungal characterized non-reduced PKSs (nrPKSs), involved in the biosynthesis of naphthopyrones, isocoumarins, ATHN, anthraquinones, dihydroxyphenylacetic acid lactones (DALs), resorcylic acid lactones (RALs), and OA and its derivatives including 3-MOA, 5-methylorsellinic acid (5-MOA), 3,5-dimethylorsellinic acid (DMOA), and orsellinic acid esters (OA esters), were collected from NCBI protein database (https://www.ncbi.nlm.nih.gov/protein). See Supplementary Table S6 for detailed sequence information. The retrieved nrPKSs library plus Preu3 and Preu6 were aligned with AlignX in Vector NTI (Lu and Moriyama, 2004), and the obtained alignment was used for the construction of a phylogenetic network using iTOL (Letunic and Bork, 2021).

### Reverse Transcription PCR Analysis

Total RNA was isolated from the mycelia of *P. isomera* XL-1326 grown on cellophane membranes covering PDA plates for 5 days at 28°C. First-strand cDNA was synthesized using the PrimeScript II First Strand cDNA Synthesis Kit (TAKARA, Shiga, Japan) according to the manufacturer's protocol. Three biological replicates were analyzed to check the expression of *pks* genes (*preu1–preu9*), and for each targeted gene, three technical replicates were performed. The RT-PCR reaction mix was prepared with 10.0 μl of DreamTaq^TM^ Green PCR Master Mix (2 × ) (Thermo Fisher Scientific, Vilnius, Lithuania), 1.0 μl of 10 μM forward primer, 1.0 μl of 10 μM reverse primer, 75 ng of cDNA, and nuclease-free water up to a total volume by 20.0 μl. The PCR protocol was as follows: initial denaturation at 95°C for 3 min, followed by 35 amplification cycles at 95°C for 30 s, 55°C for 30 s, and 72°C for 1.5 min with a final extension step at 72°C for 7 min using T100 Thermal Cycler (Bio-Rad, Hercules, CA, USA). β-actin was used as the reference gene, and PCR from genomic DNA was utilized for comparison. The used primers are listed in Supplementary Table S1.

### Heterologous Expression Constructs of the Native PKSs and the Subunit Heterocombinations

**Figure 2:**
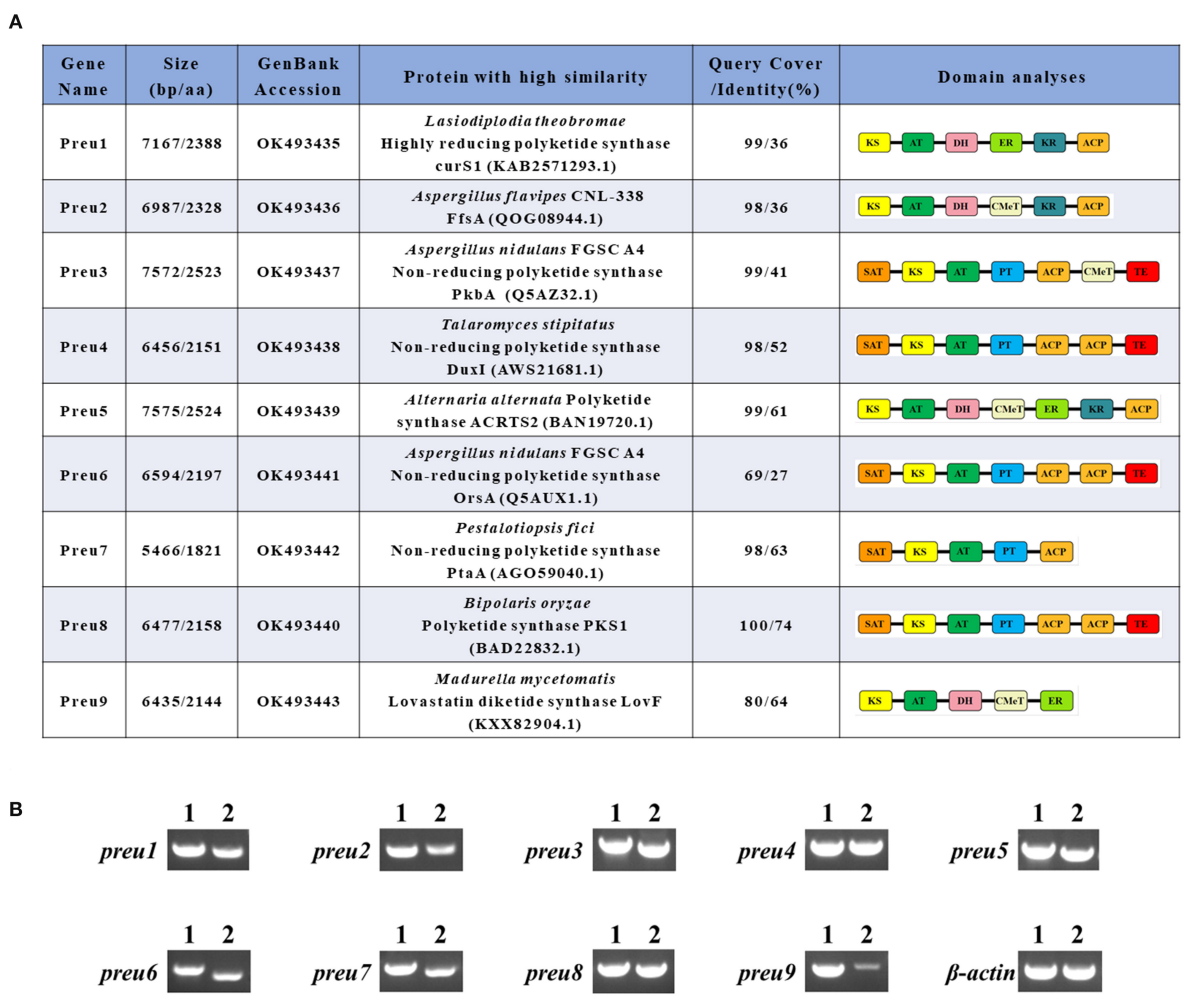
Bioinformatic and transcriptomic analysis of PKSs Preu1–Preu9 from *P. isomera* XL-1326. **(A)** Annotation of Preu1–Preu9 and their percent identities with other characterized PKSs in the specific biosynthetic gene clusters. **(B)** RT-PCR analysis (2) of the genes *preu1*–*preu9* in the *P. isomera* XL-1326 strain cultivated on PDA plates for 5 days at 28°C. PCR products from genomic DNA (1) are shown for comparison. Three biological replicates were analyzed, and for each targeted gene, three technical replicates were performed. β-actin was used as the reference gene. The sizes of the corresponding PCR products are listed in Supplementary Table S1.

Preu3, Preu4, Preu6, and Preu8 possess complete sets of synthetic and release domains for PK biosynthesis (Figure 2A); however, the Preu8 gene cluster shows 100% similarity with the melanin gene cluster (Moriwaki et al., 2004) through antiSMASH (version 5.0.0, Blin et al., 2019) analysis. Therefore, we planned to identify the functions of Preu3, Preu4, and Preu6 *via* heterologous expression in *S. cerevisiae*.

To construct the gene expression vector, the *preu3* gene was divided into three fragments amplified with the primer pairs Preu3-1F/Preu3-1R, Preu3-2F/Preu3-2R, and Preu3-3F/Preu3-3R (Supplementary Table S2), respectively, using cDNA of *P. isomera* XL-1326 as a template. The PCR products were purified with a GeneJET Gel Extraction Kit (Thermo Fisher Scientific, Vilnius, Lithuania). The three amplicons and the *Nde* I/*Pme* I fragment of YEpADH2p-FLAG-URA (*S. cerevisiae* empty expression vector; Wang et al., 2019, 2020) were linked together end-to-end with the SE Seamless Cloning and Assembly Kit (ZOMANBIO, Beijing, China) according to homologous recombination principle. The recombination reaction mix (10.0 μl) was prepared with 2.0 μl of SE cloning buffer (5 × ), 2.0 μl of fragments 1, 2, and 3, respectively, 1.0 μl of *Nde* I/*Pme* I fragment of YEpADH2p-FLAG-URA, and 1.0 μl of SE recombinase, and then proceeded to the ligation step at 37°C for 0.5 h to generate plasmid YEpPreu3. Analogous strategies were used to generate the YEpPreu4 and YEpPreu6 plasmids for the expression of *preu4* and *preu6*, respectively, using primers listed in Supplementary Table S2.

Due to the particularity and associativity of the products yielded by Preu3 and Preu6, we expected to design two new “non-native” PKSs with TE domain swapping, Preu3-TE_Preu6_ and Preu6-TE_Preu3_, to preliminarily explore the mechanism involved in the release of the products. Homologous recombination strategy analogous to YEpPreu3 generation was also used to construct the expression vectors for Preu3-TE_Preu6_ and Preu6-TE_Preu3_: for YEpPreu3-TE_Preu6_, the total five fragments are fragments 1 and 2 of Preu3, fragment 3 (amplified with the primer pairs Preu3-3F/Preu3-CMeT-R), fragment 4 (amplified with the primer pairs Preu6-TE-F/Preu6-3R), and the *Nde* I/*Pme* I fragment of YEpADH2p-FLAG-URA; and for YEpPreu6-TE_Preu3_, the total three fragments are fragment 1 (amplified with the primer pairs Preu6-BglII-F/Preu6-ACP-R), fragment 2 (amplified with the primer pairs Preu3-TE-F2/Preu3-3R), and the *Bgl* II/*Pme* I fragment of YEpPreu6 (11.0 kb). The used primers are listed in Supplementary Table S2.

### Production and Chemical Characterization of PKs

*Saccharomyces cerevisiae* BJ5464-NpgA (*MAT*α *ura3*-*52 his3*-Δ*200 leu2*-Δ*1 trp1 pep4*::*HIS3 prb1* Δ*1.6R can1 GAL*) (Ma et al., 2009) served as the host for the expression of the PKs. The heterologous expression plasmids harboring the *pks* gene were transformed into the cells of BJ5464-NpgA using the LiAc-PEG4000 procedure (Gietz and Schiestl, 2007), respectively, and the transformants were selected on synthetic complete agar plates [0.67% (wt/vol) yeast nitrogen base, 2% (wt/vol) glucose, 2% (wt/vol) agar, and 0.77 g/L Ura DropOut supplement (Clontech, Mountain View, CA, USA)]. Fermentation and extraction of PKs followed previous protocols (Wang et al., 2019, 2020). We observed the production process at the time points of 36 h, 48 h, 60 h, 72 h, and 84 h after adding the YPD medium [1% (wt/vol) yeast extract, 2% (wt/vol) peptone, and 1% (wt/vol) glucose]. Triplicate cultivations were conducted for each strain at each time point. Significance analysis was performed between 72 h and 84 h using the one-way ANOVA test, and the significance level was set as 0.05. In addition, to test the stability of PK production, eight independent *S. cerevisiae* transformants were counted for the peak area of the corresponding products after adding the YPD medium for 72 h. We determined that the yield is stable if the coefficient of variation (CV) is lower than 15%. The extracts were routinely analyzed on a Thermo Scientific UltiMate 3000 Ultra-High-Performance Liquid Chromatography (UHPLC) system, fitted with a Sunniest PFP and C18 column (250 mm × 4.6 mm, 5.0 μm, ChromaNik Technologies, Osaka, Japan). The mobile phases consisted of water (A) and methanol (B, with 0.1% acetic acid), and the flow rate was kept at 1.0 ml/min. The system was run with the following gradient program: from 5% B to 100% B for 40 min, then kept at 100% B for 5 min, and then the column was balanced with 5% B for 4 min before each sample injection. Absorbance was monitored with an UltiMate 3000 DAD Detector at 210, 254, 270, and 300 nm wavelengths.

Scaled-up cultivation of yeast strains followed previous protocols but with extraction at 72 h after adding the YPD medium (Wang et al., 2019, 2020). For isolation of the PK products, the crude extracts were first subjected to OctaDecylSilyl (ODS) column chromatography and eluted using a gradient of methanol/water. The resulting fractions containing the targets were subsequently purified by semi-preparative HPLC on an Agilent 1260 Infinity II system by eluting with MeOH/H_2_O [(0–16 min, 45:55, v/v) to yield compound **1**, *t*_R_=12.5 min; (0–12 min, 80:20, v/v) to yield compound **2**, *t*_R_=8.3 min; (0–10 min, 65:35, v/v) to yield compound **3**, *t*_R_ = 6.2 min]. Finally, the target fractions were dried *in vacuo* to get the purified PK products. The ratio of the isolated amount of products to the volume of fermentation broth is defined as the isolated yield (mg/L). UHPLC–High-resolution electrospray ionization mass spectrometry (HRESIMS) was performed on Thermo Scientific UltiMate 3000 UHPLC coupled with a Thermo Scientific Q-Exactive instrument operated in positive and negative ion modes using a capillary voltage of 3.8 kV. ^1^H and ^13^C NMR spectra were recorded on a Bruker Avance III^TM^ 600 MHz spectrometer in CD_3_OD solvent. See Supplementary Material for details.

### Synthesis of Methyl 3-Methylorsellinate (4) With 3-MOA (3) as the Substrate

Potassium carbonate (K_2_CO_3_, 3.8 mg, 0.027 mmol) was added to a solution of 3-MOA **3** (10 mg, 0.055 mmol) in acetone (0.5 ml) at 25°C, and the resulting mixture was stirred for 2 h. Then MeI (78 mg, 0.55 mmol) was added, and the resulting mixture was stirred for 12 h at 25°C (Nicolaou et al., 2000). After the reaction, acetone was removed by vacuum distillation, and the residue was purified by flash column chromatography fitted with silica gel (200–300 mesh) using n-hexane/ethyl acetate (10:1) as the mobile phase. ^1^H and ^13^C NMR spectra were recorded in CDCl_3_ solvent. See Supplementary Material for details.

### Biological Assays

OA derivatives **1**–**4** were evaluated for their antibacterial activities against eight kinds of antibiotic-resistant bacteria, including carbapenems-resistant *Acinetobacter baumannii*, carbapenems-resistant *Escherichia coli*, carbapenems-resistant *Klebsiella pneumoniae*, carbapenems-resistant *Pseudomonas aeruginosa*, methicillin-resistant *Staphylococcus aureus*, multidrug-resistant *Enterococcus faecalis*, multidrug-resistant *Enterococcus faecium*, and multidrug-resistant *Staphylococcus epidermidis*. Samples were detected in the sterile 96-well plates by the modified broth dilution test as described previously (Wu et al., 2017). Medium containing 1% DMSO and ciprofloxacin were used as the negative and positive control, respectively. Multiskan^TM^ FC Microplate Photometer (Thermo Scientific) was used to detect the cells' optical density (OD) at the wavelength of 620 nm. Triplicate cultivations were conducted for each condition. The proliferation inhibition rate was calculated based on the formula: inhibition rate = (1 – OD_620nm_ of the treated sample/OD_620nm_ of the negative control) × 100%.

Orsellinic acid derivatives **1**–**4** were also evaluated for their antifungal activities against seven agricultural pathogenic fungi: *Alternaria fragriae, Botryospuaeria berengeriana, Fusarium oxysporum* f.sp.Vasinfectum, *Helminthosporium maydis, Rhizoctonia solani, Sclerotinia sclerotiorum*, and *Verticillium dahliae* Kleb. Modified antifungal bioassays were conducted as described previously (Wu et al., 2017). Media containing 1% DMSO and ketoconazole were used as the negative and positive controls, respectively.

### Accession Numbers

The polyketide synthase genes *preu1*–*preu9* of *P. isomera* have been assigned GenBank accession numbers OK493435–OK493443, respectively (Figure 2A).

## Results

### Bioinformatic Analysis of the Nine Transcriptionally Active PKSs

The genome of *P. isomera* XL-1326 contains 40,220,700 nucleotides with 53.29% GC content (guanine-cytosine content), and the total number of genes is 10,949. According to the anti-SMASH [version 5.0.0, a software to rapidly and reliably pinpoint all the potential gene clusters for secondary metabolites (SMs), Blin et al., 2019] analysis, 21 SM gene clusters relevant to polyketide, non-ribosomal peptide, terpene, and indole synthesis, respectively, were identified, of which nine were polyketide synthase (PKS) gene clusters. The corresponding PKSs are named Preu1–Preu9, and except for *preu9*, the other eight *pks* genes were strongly transcribed in *P. isomera* XL-1326 (Figure 2B).

According to the anti-SMASH analysis, Preu1–Preu3, Preu6, and Preu9 gene clusters showed no obvious similarities with the known ones. Sequence prediction by SoftBerry's FGENESH program revealed that the *preu1*–*preu3, preu6*, and *preu9* genes consisted of an open reading frame (ORF) of lengths 7,167, 6,987, 7,572, 6,594, and 6,435 nucleotides, respectively. A database search with Pfam 27.0 and CD-Search programs showed Preu1, Preu2, and Preu9 belong to hrPKSs because they possess KR, DH, or ER domains, while Preu3 (composed of SAT-KS-AT-PT-ACP-CMeT-TE) and Preu6 (composed of SAT-KS-AT-PT-ACP-ACP-TE) are part of nrPKSs (Figure 2A). A database search with NCBI-BLASTP revealed that the deduced 2,388-amino acid sequence encoded by *preu1* shared 36% identity with the hrPKS CurS1 (LtLasS1 in Xu Y et al., 2014) of *Lasiodiplodia theobromae* (Félix et al., 2019), which is responsible for the synthesis of a pentaketide precursor during the biosynthesis of the phytotoxic fungal polyketide lasiodiplodin. The deduced 2,328 amino acid sequence encoded by *preu2* shared 36% identity with the FfsA of *Aspergillus flavipes* CNL-338, which is responsible for the biosynthesis of the cytotoxic molecule cytochalasans (Heard et al., 2020). Preu3, encoding a 2,523 amino acid sequence, is an ortholog of PkbA (41% identity), which has a confirmed role in the formation of 3-methylorsellinic acid during the biosynthesis of cichorine (Sanchez et al., 2012). Preu6, encoding a 2,197 amino acid sequence, is an ortholog of OrsA (27% identity), which is responsible for the synthesis of OA (Sanchez et al., 2010). The deduced 2,144 amino acid sequence encoded by *preu9* is an incomplete ortholog of LovF (64% identity), which is responsible for the biosynthesis of lovastatin (Xie et al., 2009).

Based on anti-SMASH, Preu4, Preu5, Preu7, and Preu8 gene clusters showed similarity with the known SM clusters to some extent. Preu5 and Preu8 gene clusters revealed 100% similarity with ACR toxin [GenBank: AB725683.1; MIBiG (Kautsar et al., 2020) accession no: BGC0001252; Izumi et al., 2012], and melanin (GenBank: AB176546.1; MIBiG accession no: BGC0001265; Moriwaki et al., 2004) gene clusters, respectively. Preu4 gene cluster revealed 28% similarity with the duclauxin (dimeric and heptacyclic fungal PKs with significant bioactivities, Gao et al., 2018) biosynthetic gene cluster from *Talaromyces stipitatus* ATCC 10500 (GenBank: EQ962653.1; MIBiG accession no: BGC0001578). Preu4, composed of 2,151 amino acids, shared 52% identity with the nrPKS DuxI. Preu7 gene cluster showed 10% similarity with the pestheic acid biosynthetic gene cluster from plant endophyte *Pestalotiopsis fici* (GenBank: KC145148.1; MIBiG accession no: BGC0000121; Xu X et al., 2014). Preu7, composed of 1,821 amino acids, shared 63% identity with the nrPKS PtaA.

### Cloning and Heterologous Expression of the PKSs With Complete Sets of Synthetic and Release Domains

**Figure 3:**
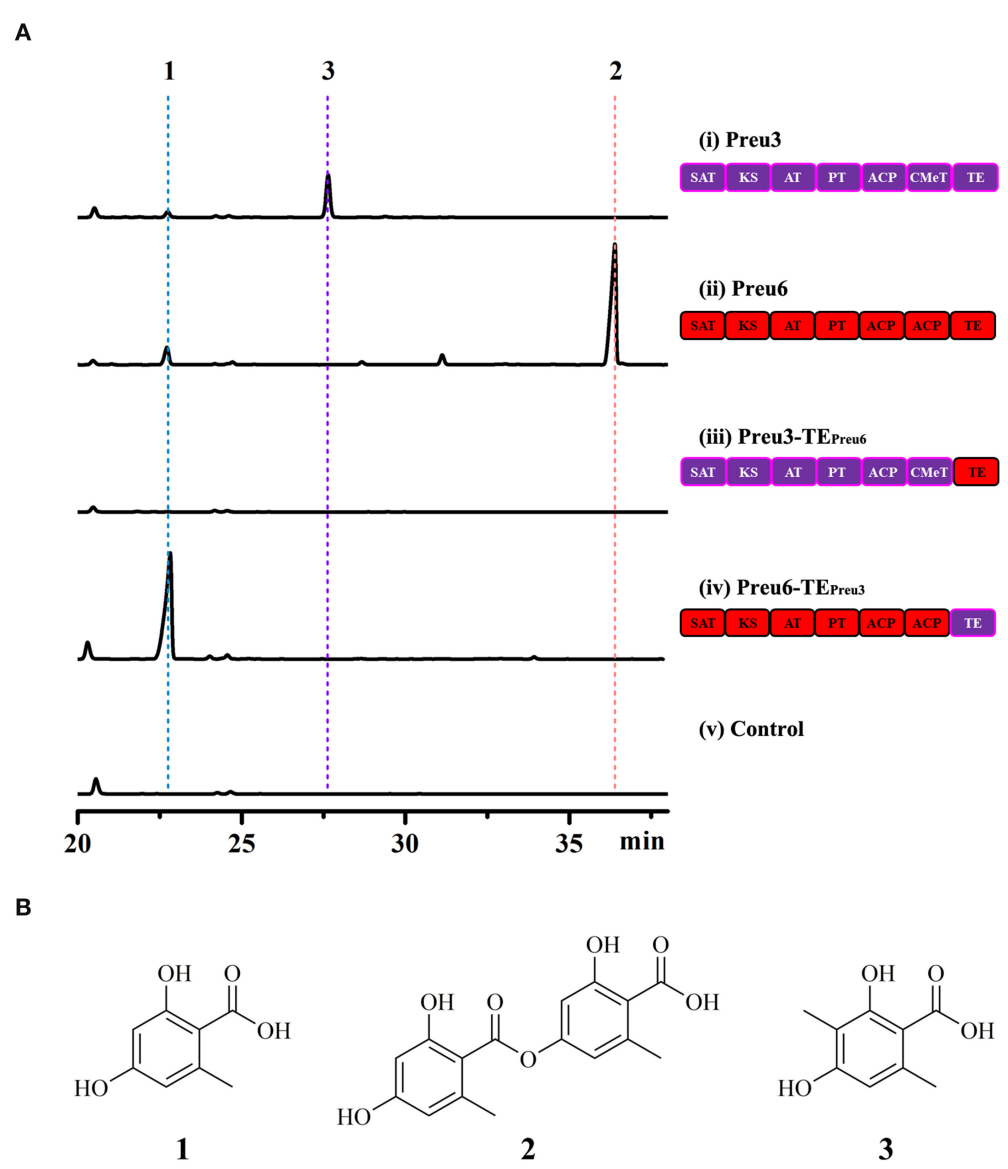
Heterologous expression of the PKSs originated from *P. isomera* XL-1326 in *S. cerevisiae* BJ5464-NpgA. **(A)** HPLC traces (DAD, 300 nm) of crude extracts of the cultures of the indicated strains cultivated for 72 h after adding the YPD medium. Control, *S. cerevisiae* BJ5464-NpgA strain with empty vector YEpADH2p-FLAG-URA. **(B)** Structures of the OA derivatives.

Based on the bioinformatic analysis, Preu3, Preu4, Preu6, and Preu8 possess complete sets of synthetic and release domains for polyketide biosynthesis (Figure 2A); however, Preu8 gene cluster shows 100% similarity with melanin gene cluster through anti-SMASH analysis. Therefore, we planned to characterize the functions of Preu3, Preu4, and Preu6 and their related products *via* heterologous expression in *S. cerevisiae*. Intron-less versions of the *preu3, preu4*, and *preu6* genes were introduced into *S. cerevisiae* BJ5464-NpgA using YEpADH2p-FLAG-URA-derived expression vectors (YEpPreu3, YEpPreu4, and YEpPreu6, which were verified by restriction enzyme digestion and sequencing), respectively. Compared to the control (BJ5464-NpgA with empty vector), Preu3 could produce a relatively high yield of 3-MOA (**3**, isolated yield: 96.2 mg/L), and Preu6 could produce a high yield of LA (**2**, isolated yield: 162.2 mg/L) (Figure 3). Isolated yield was determined at the point of scaled-up fermentation for 72 h after adding the YPD medium according to the production kinetics, which showed no significant difference in the yield between 72 h and 84 h (*P* > 0.05, Supplementary Figure S1). In addition, the stability test revealed that Preu3 and Preu6 transformants could steadily produce PKs **3** or **2**, respectively (with a CV of 10.35% and 9.03%, respectively; Supplementary Figures S2, S3). Minor quantities of OA **1** were also observed in both fermentations, which could be proved to be released as the by-product by TE_Preu3_ in Preu3 or to be released without TE_Preu6_ in Preu6 (Supplementary Figure S4). The HRESIMS and ^1^H, ^13^C-NMR spectra of compounds **1**–**3** are listed in the Supplementary Figures S6–S11 and Supplementary Tables S3–S5. Preu4 could produce two major yellow-colored compounds, **5** (m/z 277.07059 [M+H]^+^, calcd for C_14_H_13_O_6_ 277.0712) and **6** (m/z 259.06009 [M+H]^+^, calcd for C_14_H_11_O_5_ 259.0606), but the structures were not stable and hard to be characterized (Supplementary Figure S12). The HRESIMS spectra of compounds **5** and **6** are listed in the Supplementary Figures S13, S14.

### Phylogenetic Analysis of OA-Related Preu3 and Preu6

**Figure 4:**
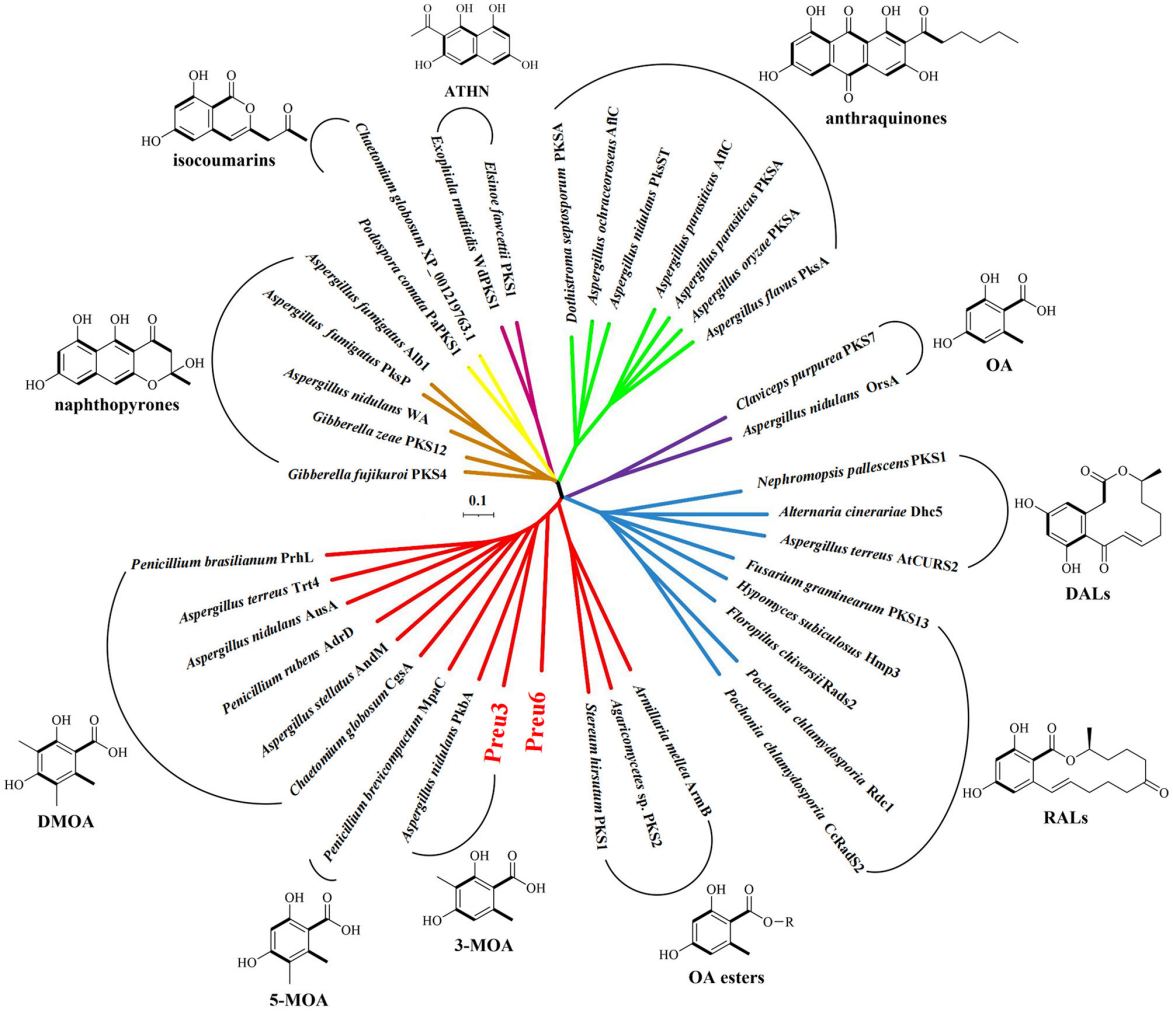
Phylogenetic network of representative fungal nrPKSs. Phylogenetic trees for the selected characterized nrPKSs, which are involved in the biosynthesis of the representative polyketides (Lackner et al., 2013; Hussain et al., 2017; Wang et al., 2020), including naphthopyrones (brown), isocoumarins (yellow), ATHN (maroon), anthraquinones (green), DALs (blue), RALs (blue), OA (purple), and its derivatives (red) covering 3-MOA, 5-MOA, DMOA, and OA esters. For accession numbers, see Supplementary Table S6.

Phylogenetic relationships have been proven to be highly informative in terms of predicting the functional or structural aspects of secondary metabolism (Ziemert and Jensen, 2012). Herein, we constructed a phylogenetic network using full sequences of the characterized nrPKSs, which are involved in the biosynthesis of the representative PKs, including naphthopyrones, isocoumarins, ATHN, anthraquinones, DALs, RALs, and OA and its derivatives, including 3-MOA, 5-MOA, DMOA, and OA esters (Lackner et al., 2013; Hussain et al., 2017; Wang et al., 2020) (Figure 4; Supplementary Table S6). The network demonstrated that the nrPKSs can be grouped into different clusters according to their corresponding product types, except for two OA synthases, OrsA from *A. nidulans* (Sanchez et al., 2010), and PKS7 from *C. purpurea* (Lünne et al., 2020), which have very low similarities to the other OA derivative-related nrPKSs. Remarkably, our Preu3 and Preu6, biosynthesizing 3-MOA **3** and LA **2**, respectively, are clustered into OA derivative synthase groups. In addition, Preu3 and PkbA, which was characterized to synthesize 3-MOA in *A. nidulans* (Sanchez et al., 2012), belong to one subclade. Thus, the construction of a phylogenetic network might be a suitable tool for the prediction of nrPKS functions from genomic data.

### The Release Form of the OA Derivatives Determined by TE_Preu3_ and TE_Preu6_

According to the previous research, TE domains act as nonequivalent decision gates determining both the shape and the yield of the polyketide products (Xu et al., 2013a; Wang et al., 2020). Due to the particularity and associativity of the OA derivatives yielded by Preu3 and Preu6, we expected to design two new “non-native” PKSs, Preu3-TE_Preu6_ and Preu6-TE_Preu3_, to preliminarily explore the mechanism involved in the synthesis of the products. As shown in Figure 3A, swapping TE_Preu3_ with TE_Preu6_, Preu3-TE_Preu6_ could not release any products, while exchanging TE_Preu6_ with TE_Preu3_, Preu6-TE_Preu3_ could produce a high yield of **1** (isolated yield: 253.4 mg/L). Isolated yield was determined at the point of scaled-up fermentation for 72 h after adding the YPD medium according to the production kinetics, which reached the peak at 72 h (Supplementary Figure S1). In addition, the stability test revealed that Preu6-TE_Preu3_ transformants could steadily produce OA **1** (with a CV of 9.76%; Supplementary Figure S5). All the results revealed that TE_Preu3_ could just release the smaller OA derivatives, **1** and **3**, while TE_Preu6_ acts as the decision gate to just release LA **2**. With TE domain swapping, we constructed a new “non-native” PKS, Preu6-TE_Preu3_ (encoding a 2,228-amino acid sequence, shared 27% identity with OrsA, Query cover 68%), which could generate a great yield of OA through heterologous expression in *S. cerevisiae* BJ5464-NpgA.

### Conversion of Compound 3 to Compound 4 by Chemical Synthesis

Methyl 3-methylorsellinate **4**, also called synthetic oak moss, is mainly responsible for the characteristic earthy-moss-like odor of the oak moss absolute, one of the eight ingredients of the fragrance mix (Bernard et al., 2003). Using a chemical synthesis strategy, 3-MOA **3**, produced by Preu3 with a relatively high yield, could be methylated (MeI/K_2_CO_3_) to generate methyl ester **4** (88%) as a white crystalline powder. As shown in Supplementary Figure S15, the chemical structure of **4** was characterized by comparing the NMR data to the literature (Wang et al., 2005).

### Antimicrobial Activities of OA Derivatives 1–4

**Figure 5:**
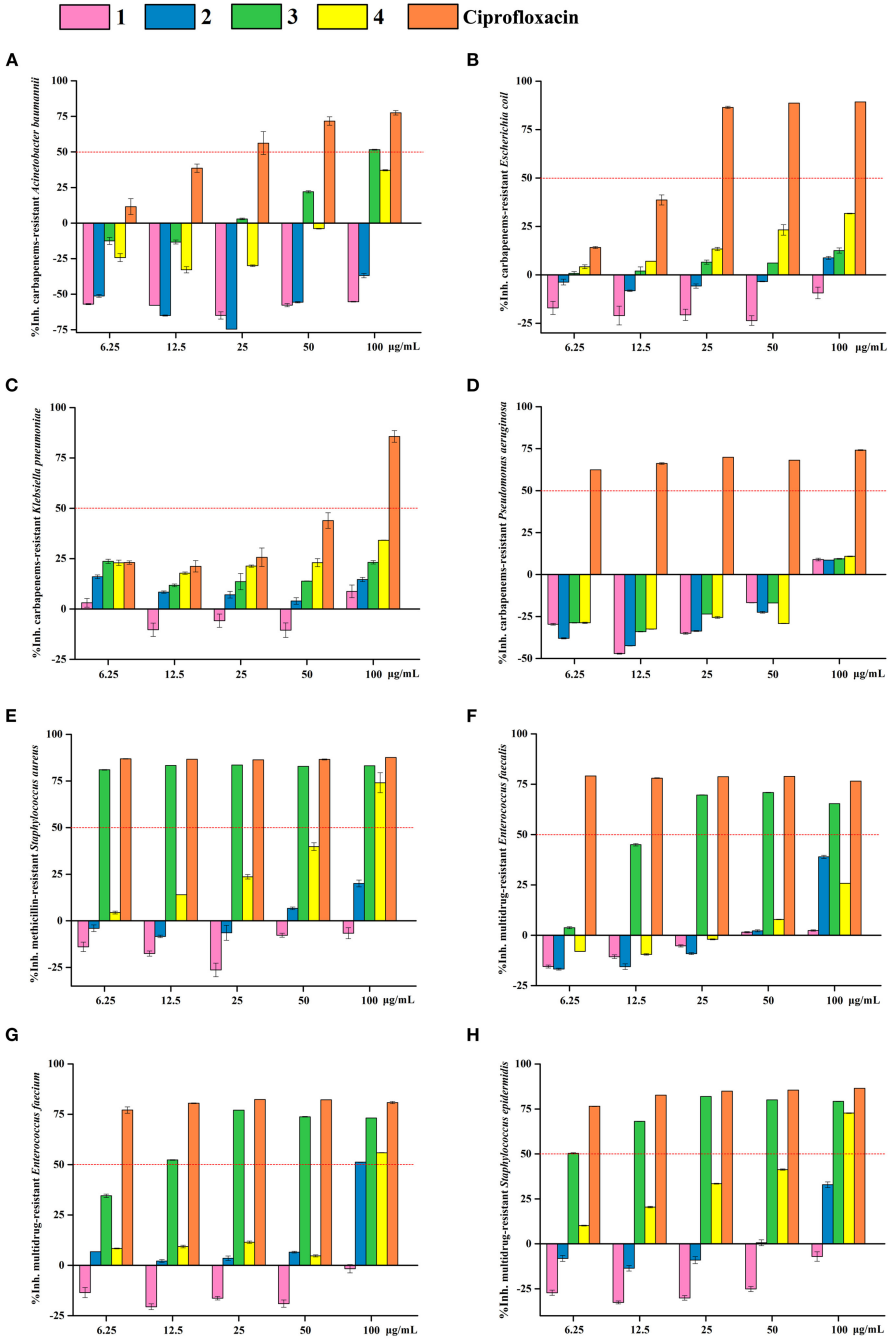
Antibacterial activity of OA derivatives **1**–**4**. The inhibitory rates of OA derivatives **1**–**4** against carbapenems-resistant *A. baumannii* **(A)**, carbapenems-resistant *E. coli* **(B)**, carbapenems-resistant *K. pneumoniae* **(C)**, carbapenems-resistant *P. aeruginosa* **(D)**, methicillin-resistant *S. aureus* **(E)**, multidrug-resistant *E. faecalis* **(F)**, multidrug-resistant *E. faecium* **(G)**, and multidrug-resistant *S. epidermidis* **(H)**. Pink columns indicate OA **1**, blue columns indicate LA **2**, green columns indicate 3-MOA **3**, and yellow columns indicate methyl 3-methylorsellinate **4**. Ciprofloxacin was used as the positive control.

OA derivatives **1**–**4** were tested for their antibacterial activities against eight antibiotic-resistant bacteria (carbapenems-resistant *A. baumannii*, carbapenems-resistant *E. coli*, carbapenems-resistant *K. pneumoniae*, carbapenems-resistant *P. aeruginosa*, methicillin-resistant *S. aureus*, multidrug-resistant *E. faecalis*, multidrug-resistant *E. faecium*, and multidrug-resistant *S. epidermidis*) and antifungal activities toward seven agricultural pathogenic fungi (*A. fragriae, B. berengeriana, F. oxysporum* f.sp.Vasinfectum, *H. maydis, R. solani, S. sclerotiorum*, and *V. dahliae* Kleb). The results revealed that 3-MOA **3** exhibited significant antibacterial activities against methicillin-resistant *S. aureus* and multidrug-resistant *S. epidermidis* with IC_50_ (the half-maximal inhibitory concentration) values <6.25 μg/ml, against multidrug-resistant *E. faecium* with an IC_50_ value around 12.5 μg/ml, and against multidrug-resistant *E. faecalis* with an IC_50_ value between 12.5 and 25 μg/ml (Figure 5). Moreover, methyl 3-methylorsellinate **4** showed moderate activity against methicillin-resistant *S. aureus*, multidrug-resistant *E. faecium*, and multidrug-resistant *S. epidermidis* with IC_50_ values between 50 and 100 μg/ml (Figure 5).

In contrast, 3-MOA **3** did not show any antifungal activity, while methyl 3-methylorsellinate **4** displayed broad-spectrum activity against all the seven agricultural pathogenic fungi with the MIC (minimum inhibitory concentrations) values of 25–50 μg/ml (Table 1). Moreover, LA **2** demonstrated significant activity against *B. berengeriana* with a MIC value of 25 μg/ml, and OA **1** exhibited moderate activity against *H. maydis* with a MIC value of 50 μg/ml (Table 1).

**Table 1:** Antifungal activity of OA derivatives **1**–**4** (MIC, μg/ml)^a^.

**Compounds**	***B. berengeriana***	***F. oxysporum*** **f.sp.Vasinfectum**	***H. maydis***	***V. dahliae*** **Kleb**	***S. sclerotiorum***	***R. solani***	***A. fragriae***
**1**	100	>100	50	100	100	>100	>100
**2**	25	>100	100	100	100	>100	>100
**3**	>100	>100	>100	>100	>100	>100	>100
**4**	50	25	50	25	50	50	50
Ketoconazole	<0.78	6.25	<0.78	<0.78	<0.78	6.25	<0.78

^a^*MIC, minimum inhibitory concentration*.

## Discussion

The genus *Preussia* is mainly isolated from soil and plant materials as endophytes, and the recent studies mostly focus on the diversity of the species, metabolites, and their bioactivities (Xu et al., 2019; Chen et al., 2020; Ameen et al., 2021). Herein, based on genome mining, we discovered nine transcriptionally active polyketide synthases (PKSs), Preu1–Preu9, of which Preu3 and Preu6 were identified to biosynthesize 3-methylorsellinic acid (3-MOA) and lecanoric acid (LA) through heterologous expression in *S. cerevisiae* BJ5464-NpgA (Ma et al., 2009), respectively (Figure 3).

Taking both the general mechanism of PKSs and our research results into account, the biosynthesis pathway of 3-MOA by Preu3 was proposed, as shown in Supplementary Figure S16 (Cox, 2007; Tippelt and Nett, 2021). The starter unit ACP transacylase (SAT) domain is responsible for capturing an acetyl-CoA (when a mutation was introduced into the SAT domain, 3-MOA could not be produced; data not shown) and transferring it to the acyl carrier protein (ACP) domain activated by phosphopantetheinyl transferase (PPTase) first, and then transferring to the ketoacyl synthase (KS) domain; the acyl transferase (AT) domain loads a malonyl-CoA and transfers it to the ACP domain. Afterward, KS mediates the intrinsic Claisen condensation between the ACP-tethered substrate and the previously formed KS-bound polyketide intermediate. This reaction is accompanied by a decarboxylation step and results in the formation of a β-ketoacyl thioester. After two rounds of extension, with the participation of *S*-adenosyl-*L*-methionine, the methyl transferase (CMeT) domain methylates the C–C skeleton to introduce methyl group (when the CMeT domain was deleted, OA rather than 3-MOA could be produced; data not shown). Finally, after another round of extension, the intermediate adopts a C2–C7 cyclization directed by the product template (PT) domain, and the release of 3-MOA from Preu3 is mediated by the thioesterase (TE) domain (when a mutation was introduced into the TE domain, 3-MOA could not be produced; Supplementary Figure S4).

However, the biosynthesis pathway of LA by Preu6 was harder to be illustrated. With TE domain swapping, Preu3-TE_Preu6_ could not release any products, while Preu6-TE_Preu3_ could produce high yield of OA (Figure 3). The results revealed that TE_Preu3_ could just release the smaller OA derivatives, OA and 3-MOA, while TE_Preu6_ acts as the decision gate to just release LA. Thus, we proposed that under the combined action of SAT-KS-AT-PT-ACP-ACP in Preu6, the OA chain forms by hanging on to the ACP domain and transfers to TE_Preu6_, and then TE_Preu6_ catalyzes two molecules of OA to form LA that is released (Supplementary Figure S17). The exact mechanism involved in the formation of LA from Preu6 remains to be studied.

*Saccharomyces cerevisiae* is one of the most widely used hosts for the production of recombinant polyketides (PKs) and non-ribosomal peptides (NRPs) (Tippelt and Nett, 2021). In this study, *S. cerevisiae* BJ5464-NpgA has been utilized for the engineered production of fungal PKs and NRPs, i.e., the semi-synthetic cholesterol-lowing medication simvastatin with a titer of 55 mg/L (Bond and Tang, 2019); the benzenediol lactones including the heat shock response modulator monocillin II with an isolated yield of 9 mg/L and the phytotoxins 10,11-dehydrocurvularin, desmethyl-lasiodiplodin, and *trans*-resorcylide with isolated yields of 6 mg/L, 10 mg/L, and 8 mg/L, respectively (Xu et al., 2013b, Xu Y et al., 2014); and the azaphilone pre-asperfuranone with an isolated yield of 5.1 mg/L (Bai et al., 2016) and the anticancer cyclo-oligomer depsipeptides beauvericins and bassianolide with titer values of 33.8 mg/L and 21.7 mg/L, respectively (Yu et al., 2013). Herein, through heterologous expression in the *S. cerevisiae* BJ5464-NpgA strain, Preu3, Preu6, and Preu6-TE_Preu3_ were found to release high yields of 3-MOA **3** (isolated yield: 96.2 mg/L), LA **2** (isolated yield: 162.2 mg/L), and OA **1** (isolated yield: 253.4 mg/L), respectively, with fermentation carried for 72 h after the addition of the YPD medium (Figure 3). According to Cayman Chemical Company (Ann Arbor, Michigan, USA), the quoted prices for OA **1**, LA **2**, and 3-MOA **3** are $44, $1,240, and $148/10 mg, respectively. Therefore, we have successfully constructed a series of OA-derived engineering strains with great commercial value, and we also provide a new platform to produce OA derivatives by environment-friendly methods.

Importantly, methyl 3-methylorsellinate **4**, also called synthetic oak moss, is mainly responsible for the characteristic earthy-moss-like odor of the oak moss absolute (Bernard et al., 2003). Oak moss is a rare lichen resource, and its extract and essential oil are widely used in various commodities, i.e., perfumes, skin care products, cigarettes, and incense sticks. In addition, Oak moss is used as a food additive in beverages, baked foods, puddings, and soft sweets. However, oak moss extract and essential oil, being a natural product, have some inherent shortcomings in market application. First, natural oak moss resources are scarce and expensive. Second, the quality of oak moss is different due to the differences in the sources of raw material and extraction processes. Finally, the mixture of natural oak moss extract and essential oil is composed of complex components, and some of them have been identified as allergens, and the EU Cosmetics Regulation has restricted the use of these allergens (Rastogi et al., 2004). Therefore, methyl 3-methylorsellinate **4**, which is the main source of the earthy-moss-like odor and could be synthesized from 3-methylorsellinic acid (3-MOA, **3**), can be developed as a substitute for oak moss (Lv et al., 2019, 2020). In this study, we herein supply a scheme to methylate 3-MOA **3** (MeI/K_2_CO_3_) to generate methyl 3-methylorsellinate **4** (88%). Interestingly, 3-MOA **3** exhibited a significant antibacterial activity but no antifungal activity, while after methylation, methyl 3-methylorsellinate **4** displayed broad-spectrum antifungal activity but weak antibacterial activity (Figure 5; Table 1).

In conclusion, the results of the current study demonstrated that we characterized three novel nrPKSs, Preu3, Preu6, and Preu6-TE_Preu3_, which are responsible for the biosynthesis of OA or its derivatives, LA and 3-MOA, based on genome mining and combinatorial biosynthesis. With the nrPKSs, we have successfully constructed a series of high-yield OA-derived engineering strains with great commercial value, and we also provide a new platform to produce OA derivatives by environment-friendly methods.

## Data Availability Statement

The datasets presented in this study can be found in online repositories. The names of the repository/repositories and accession number(s) can be found in the article/Supplementary Material.

## Author Contributions

DZ performed the experiments and contributed to the data analysis. YX contributed to biological assays and compound isolation. SG contributed to phylogenetic analysis. YG contributed to production kinetics. XC contributed to text correction. MY contributed to chemical synthesis. XY contributed to manuscript editing. QL designed the project, analyzed the data, wrote, and edited the manuscript. All the authors have read and approved the manuscript prior to submission.

## Conflict of Interest

The authors declare that the research was conducted in the absence of any commercial or financial relationships that could be construed as a potential conflict of interest.

## Publisher's Note

All claims expressed in this article are solely those of the authors and do not necessarily represent those of their affiliated organizations, or those of the publisher, the editors and the reviewers. Any product that may be evaluated in this article, or claim that may be made by its manufacturer, is not guaranteed or endorsed by the publisher.
