# Supplementary material for: Cloning and Functional Characterization of the Polyketide Synthases Based on Genome Mining of Preussia isomera XL-1326
Source: Front Microbiol. 2022 May 4;13:819086. doi: 10.3389/fmicb.2022.819086 (PMC9116485; doi:10.3389/fmicb.2022.819086)
Supplement: Supplementary file 1 [file Data_Sheet_1.docx]

Supplementary Material for *Frontiers in Microbiology*.

**Cloning and functional characterization of the polyketide synthases based on genome mining of *Preussia isomera* XL-1326**

Qingpei Liu*^1#,^**, Dan Zhang*^1#^*, Yao Xu*^1^*, Shuaibiao Gao*^1^*, Yifu Gong*^1^*, Xianhua Cai*^1^*, Ming Yao*^1^**, and Xiaolong Yang*^1^**

*^1^* The Modernization Engineering Technology Research Center of Ethnic Minority Medicine of Hubei Province, School of Pharmaceutical Sciences, South-Central University for Nationalities, Wuhan, Hubei Province, 430074, P.R. China

*^#^* These authors contributed equally to this work.

*** Corresponding authors: Qingpei Liu, E-mail: lqp_2019@scuec.edu.cn; Ming Yao, E-mail: yaoming536891@163.com; and Xiaolong Yang, E-mail: yxl19830915@163.com

Contents

[Table S1. Primers for RT-PCR analysis of the *pks* genes S1](#_Toc94261576)

[Table S2. Primers used for the gene heterologous expression S2](#_Toc94261577)

[Figure S1. Production kinetics (DAD, 300 nm) of the PKs in the indicated strains S3](#_Toc94261578)

[Figure S2. HPLC traces (DAD, 300 nm) of crude extracts of different Preu3 transformants S4](#_Toc94261579)

[Figure S3. HPLC traces (DAD, 300 nm) of crude extracts of different Preu6 transformants S5](#_Toc94261580)

[Figure S4. HPLC traces (DAD, 300 nm) of crude extracts of Preu3, Preu6 and their TE mutants S6](#_Toc94261582)

[Figure S5. HPLC traces (DAD, 300 nm) of crude extracts of different Preu6-TE_Preu3_ transformants S7](#_Toc94261584)

[Figure S6. The HRESIMS spectra of compound 1 (orsellinic acid) S8](#_Toc94261586)

[Table S3. ^1^H (500 MHz) and ^13^C (125 MHz) NMR data for 1 in methanol-*d_4_* (*δ* in ppm) S8](#_Toc94261587)

[Figure S7. The ^1^H (500 MHz), ^13^C-NMR (125 MHz) spectra of compound 1 in methanol-*d_4_* S9](#_Toc94261588)

[Figure S8. The HRESIMS spectra of compound 2 (lecanoric acid) S10](#_Toc94261589)

[Table S4. ^1^H (500 MHz) and ^13^C (125 MHz) NMR data for 2 in methanol-*d_4_* (*δ* in ppm, *J* in Hz) S11](#_Toc94261590)

[Figure S9. The ^1^H (500 MHz), ^13^C-NMR (125 MHz) spectra of compound 2 in methanol-*d_4_* S12](#_Toc94261591)

[Figure S10. The HRESIMS spectra of compound 3 (3-methylorsellinic acid) S13](#_Toc94261592)

[Table S5. ^1^H (500 MHz) and ^13^C (125 MHz) NMR data for 3 in methanol-*d_4_* (*δ* in ppm) S13](#_Toc94261593)

[Figure S11. The ^1^H (500 MHz), ^13^C-NMR (125 MHz) spectra of compound 3 in methanol-*d_4_* S14](#_Toc94261594)

[Figure S12. HPLC traces (DAD, 300 nm) of crude extracts of Preu4 S15](#_Toc94261595)

[Figure S13. The HRESIMS spectra of compound 5 S16](#_Toc94261596)

[Figure S14. The HRESIMS spectra of compound 6 S17](#_Toc94261597)

[Figure S15. The ^1^H (500 MHz), ^13^C-NMR (125 MHz) spectra of compound 4 in chloroform-*d_4_* S18](#_Toc94261598)

[Figure S16. Proposed mechanism of Preu3 S19](#_Toc94261599)

[Figure S17. Proposed mechanism of Preu6 S20](#_Toc94261600)

[Table S6. Sequence information for the fungal nrPKSs S21](#_Toc94261601)

[Amino acid sequences of polyketide synthases Preu1-Preu9 S24](#_Toc94261602)

[References S33](#_Toc94261603)

**Table S1. Primers for RT-PCR analysis of the *pks* genes**

| **Name** | **Sequence (5′→3′)** | **PCR product (bp)**  (cDNA as template) | **PCR product (bp)**  (gDNA as template) |
| --- | --- | --- | --- |
| Preu1F | TCCGCACTCGAACAAGTCATAACCGTC | 835 | 884 |
| Preu1R | CTGATTGGAGCAGTGCGTCTTGAAACT |  |  |
| Preu2F | TCCTGGCTACCGCAAAGGATAAGTT | 658 | 658 |
| Preu2R | AGTTCAGCAAGGCAGAGCACAGAGT |  |  |
| Preu3F | TTGCTGGACGGATGGTGGCTCTTTGAAGAC | 1263 | 1377 |
| Preu3R | CTAAGCATTCCGACACTGCTCCACCAACCACT |  |  |
| Preu4F | GAAAACGAGGGTACAGGGCTAAGAGTC | 893 | 893 |
| Preu4R | TCATCCCTGCAAAGCACGTGCAATG |  |  |
| Preu5F | GTCACATTGGGCAAGGATGAGAAAGTC | 1161 | 1224 |
| Preu5R | TCAAATAACCCTCTGGCTCAGAATATCCGTCGCC |  |  |
| Preu6F | TTCAACGCGTCTCTCCTATGCCCTT | 908 | 1014 |
| Preu6R | TCAAGCGTTCTGAATCAAAAAGTCAAGCCCCG |  |  |
| Preu7F | ATTTGGACCGGGACGCATCAACTAT | 985 | 1093 |
| Preu7R | GTCCGGGTTCGCTTCAAGATAGGCTAT |  |  |
| Preu8F | CACTGGCATCGATCTACCGTCAACTTTC | 1000 | 1054 |
| Preu8R | CTATAGCTTGAGACCCTCCTTGAGATG |  |  |
| Preu9F | ATTGCACGCCTTCGTAAGGGACAATC | 885 | 885 |
| Preu9R | CTAGTGGGAGGAAAGGTCCTTGATGACT |  |  |
| *β-*actinF | GTATCATGATTGGTATGGGACAG | 543 | 543 |
| *β-*actinR | AGTCGAGGGCGACGTAGCAGAGCT |  |  |

# Table S2. Primers used for the gene heterologous expression

| **Name** | **Sequence (5′→3′)** | **Description of the amplicon (length)** |
| --- | --- | --- |
| Preu3-1F | GACTACAAAGACGATGACGACAAGCTTCATATGAACCCTCCTAGCGCTTTGGCCTTTGG | Fragment 1 of the *preu3* gene (2492 bp) |
| Preu3-1R | GCAATGGCCCGACAATTGGCTTCCACCGCATCAGTGCCATCTCCGGATATAAAC |  |
| Preu3-2F | GGTGGAAGCCAATTGTCGGGCCATTGCCAAAT | Fragment 2 of the *preu3* gene (2554 bp) |
| Preu3-2R | CGAATCAATGCCCAGATCGTCAAGCGT |  |
| Preu3-3F | ACGCTTGACGATCTGGGCATTGATTCGCTTATGGTCATGGAGGTGCAAACTGAG | Fragment 3 of the *preu3* gene (2567 bp) |
| Preu3-3R | AGTGATGGTGATGGTGATGTCCGTTTAAACCTAAGCATTCCGACACTGCTCCACCAAC |  |
| Preu4-1F | GACTACAAAGACGATGACGACAAGCTTCATATGGAGGCAGTTGTTTCATCGGCACCATTC | Fragment 1 of the *preu4* gene (2223 bp) |
| Preu4-1R | GTGGACATTCCGGTCTGCCAAATCTGATGGGTAGTTACGGTTGAGCTTGC |  |
| Preu4-2F | CCCATCAGATTTGGCAGACCGGAATGTCCACATTCCT | Fragment 2 of the *preu4* gene (2042 bp) |
| Preu4-2R | CAGATATGCGTTGCGCTTCCATTCAG |  |
| Preu4-3F | TACTGAATGGAAGCGCAACGCATATCTGATTTCCAGCCGCATCGCATCGCTCCACAAG | Fragment 3 of the *preu4* gene (2165 bp) |
| Preu4-3R | AGTGATGGTGATGGTGATGTCCGTTTAAACTCATCCCTGCAAAGCACGTGCAATGCGAG |  |
| Preu6-1F | GACTACAAAGACGATGACGACAAGCTTCATATGTCTAATTCTACACGTGACTATCCCATA | Fragment 1 of the *preu6* gene (2218 bp) |
| Preu6-1R | CATGTTTGATCATTGCAAGTACTTTCAGCAGACCAGCGACGCCAGCGCCAGT |  |
| Preu6-2F | GCTGAAAGTACTTGCAATGATCAAACATGGAGGTATCCCGCCT | Fragment 2 of the *preu6* gene (2153 bp) |
| Preu6-2R | TCGGCTTGAGGAATTCCGAATAGGT |  |
| Preu6-3F | TCATGACCTATTCGGAATTCCTCAAGCCGATTTCGTCGATCATCCTGCGCGAAAAC | Fragment 3 of the *preu6* gene (2272 bp) |
| Preu6-3R | AGTGATGGTGATGGTGATGTCCGTTTAAACTCAAGCGTTCTGAATCAAAAAGTCAAGCC |  |
| Preu3-CMeT-R | CGCAGATGCTCCCAGCTGTTGCTCGAGCGCTCCGTCTTCAAAGAGCCACCAT | Paired with primer Preu3-3F to generate fragment 3 of the *preu3*-*TE_Preu6_* gene (1380 bp) |
| Preu6-TE-F | CTCGAGCAACAGCTGGGAGCATCTG | Paired with primer Preu6-3R to generate fragment 4 of the *preu3*-*TE_Preu6_* gene (1188 bp) |
| Preu6-BglII-F | GATGACAGGCGTGAGGTTCAACAAGATGGAGATCTCAAAGTTAGCGAAATCGCTCTCG | Fragment 1 of the *preu6*-*TE_Preu3_* gene (751 bp) |
| Preu6-ACP-R | AGATAAGGTCATGTCTGCAACAACAGCGG |  |
| Preu3-TE-F2 | TCCGCTGTTGTTGCAGACATGACCTTATCTCGACCACACGTTCTTGCTGACGAAAAT | Paired with primer Preu3-3R to generate fragment 2 of the *preu6*-*TE_Preu3_* gene (1290 bp) |

# Figure S1. Production kinetics (DAD, 300 nm) of the PKs in the indicated strains


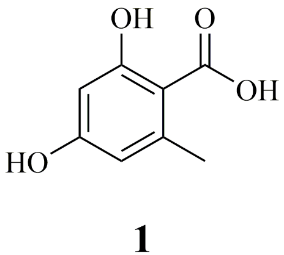

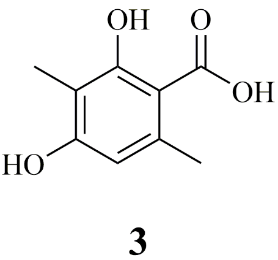

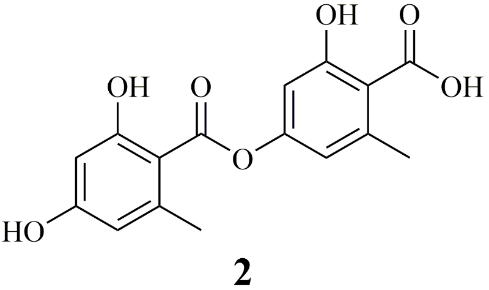

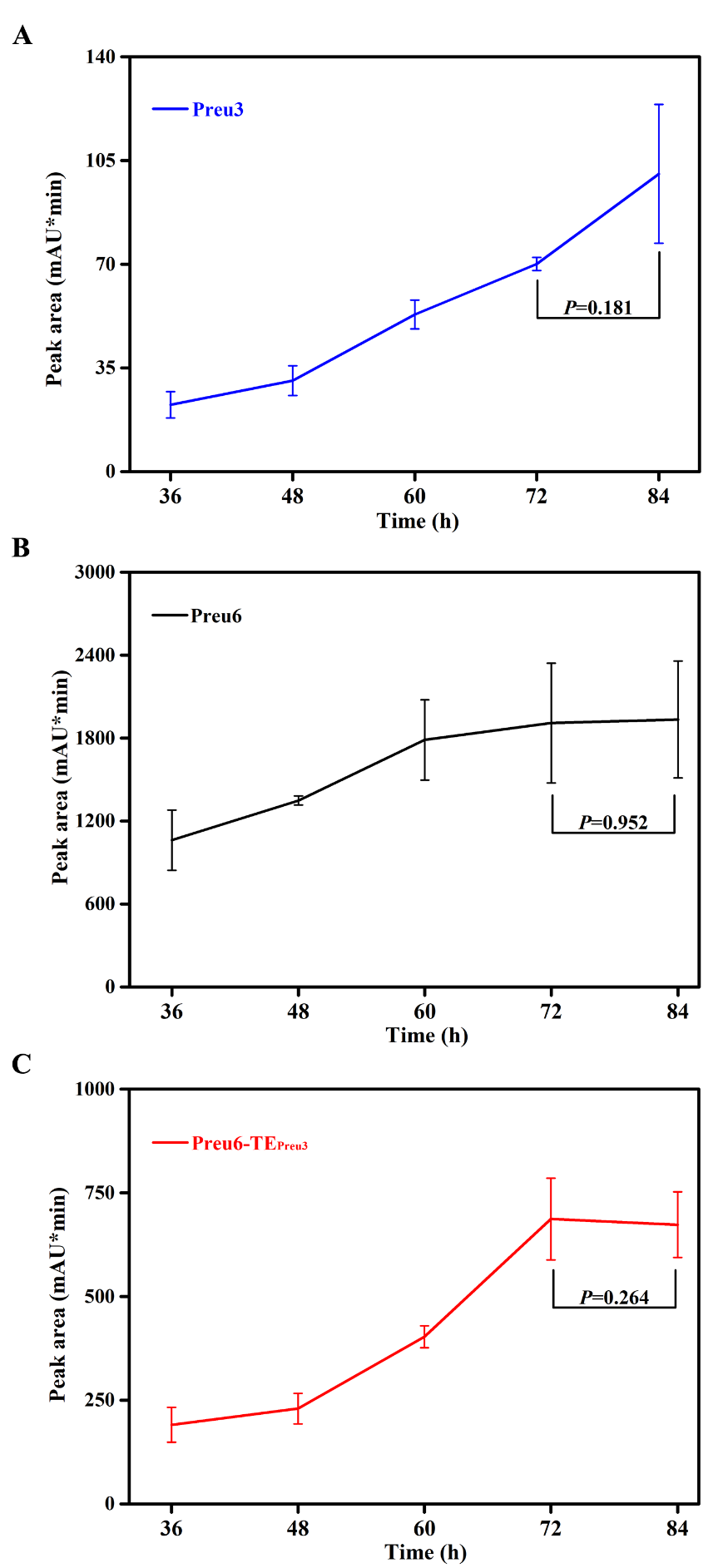


# Figure S2. HPLC traces (DAD, 300 nm) of crude extracts of different Preu3 transformants





Data analysis: Mean (MN) = 77.49; Standard Deviation (SD) = 8.02; Coefficient of Variation (CV) = (SD/MN) × 100% = 10.35%

# Figure S3. HPLC traces (DAD, 300 nm) of crude extracts of different Preu6 transformants

#



Data analysis: Mean (MN) = 1863.35; Standard Deviation (SD) = 168.24; Coefficient of Variation (CV) = (SD/MN) × 100% = 9.03%

# Figure S4. HPLC traces (DAD, 300 nm) of crude extracts of Preu3, Preu6 and their TE mutants

#
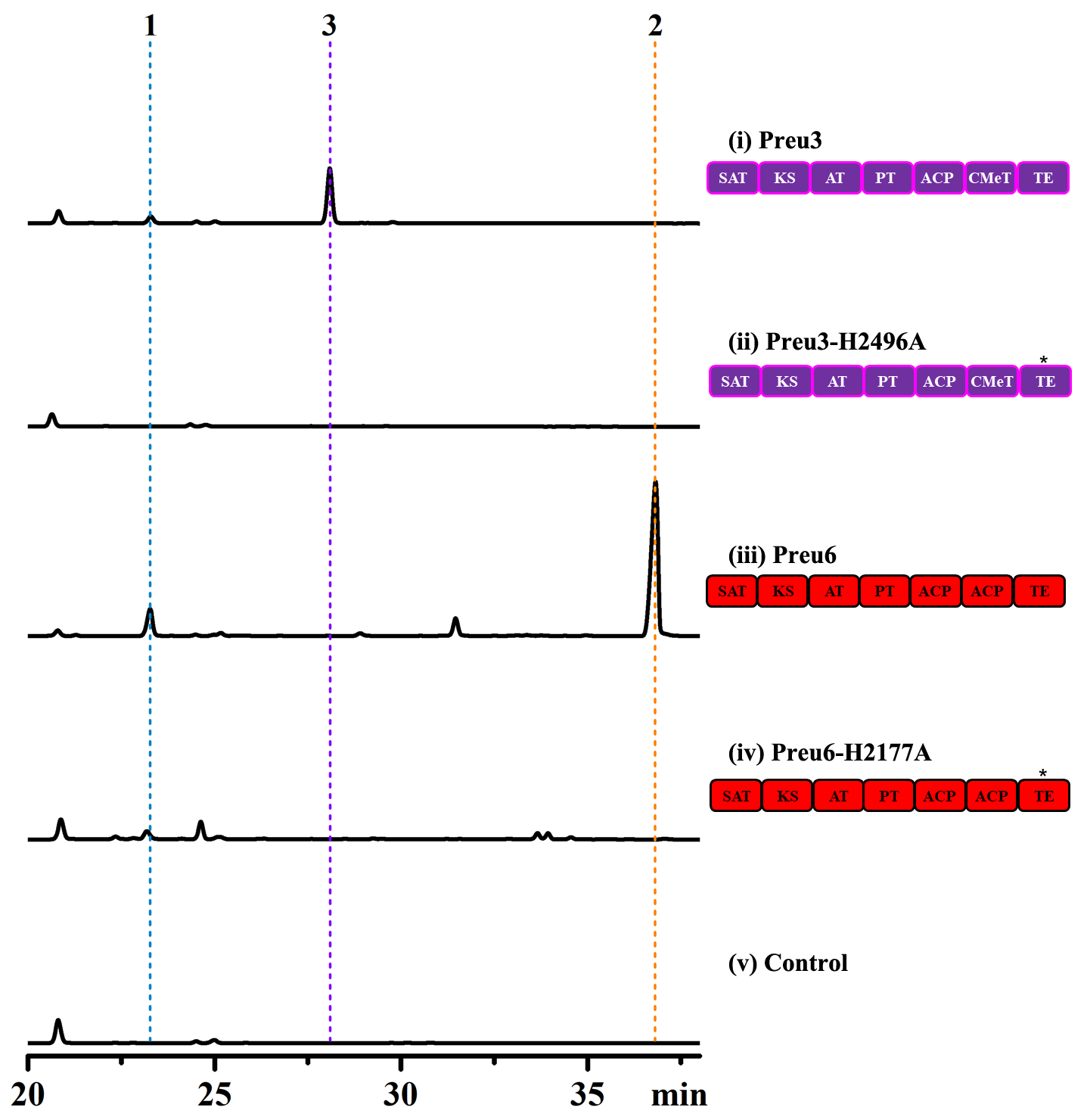


# Figure S5. HPLC traces (DAD, 300 nm) of crude extracts of different Preu6-TE_Preu3_ transformants

#



Data analysis: Mean (MN) = 679.88; Standard Deviation (SD) = 66.38; Coefficient of Variation (CV) = (SD/MN) × 100% = 9.76%

# Figure S6. The HRESIMS spectra of compound 1 (orsellinic acid)





[M+H]+

[M-H]-

# Table S3. ^1^H (500 MHz) and ^13^C (125 MHz) NMR data for 1 in methanol-*d_4_* (*δ* in ppm)

| No. | δC | δH (m, area) |
| --- | --- | --- |
| 1 | 112.1, C |  |
| 2 | 166.7, C |  |
| 3 | 101.6, CH | 6.10 (s, 1H) |
| 4 | 163.3, C |  |
| 5 | 112.1, CH | 6.15 (s, 1H) |
| 6 | 145.4, C |  |
| 7 | 175.8, C |  |
| 8 | 24.4, CH_3_ | 2.48 (s, 3H) |

# Figure S7. The ^1^H (500 MHz), ^13^C-NMR (125 MHz) spectra of compound 1 in methanol-*d_4_*


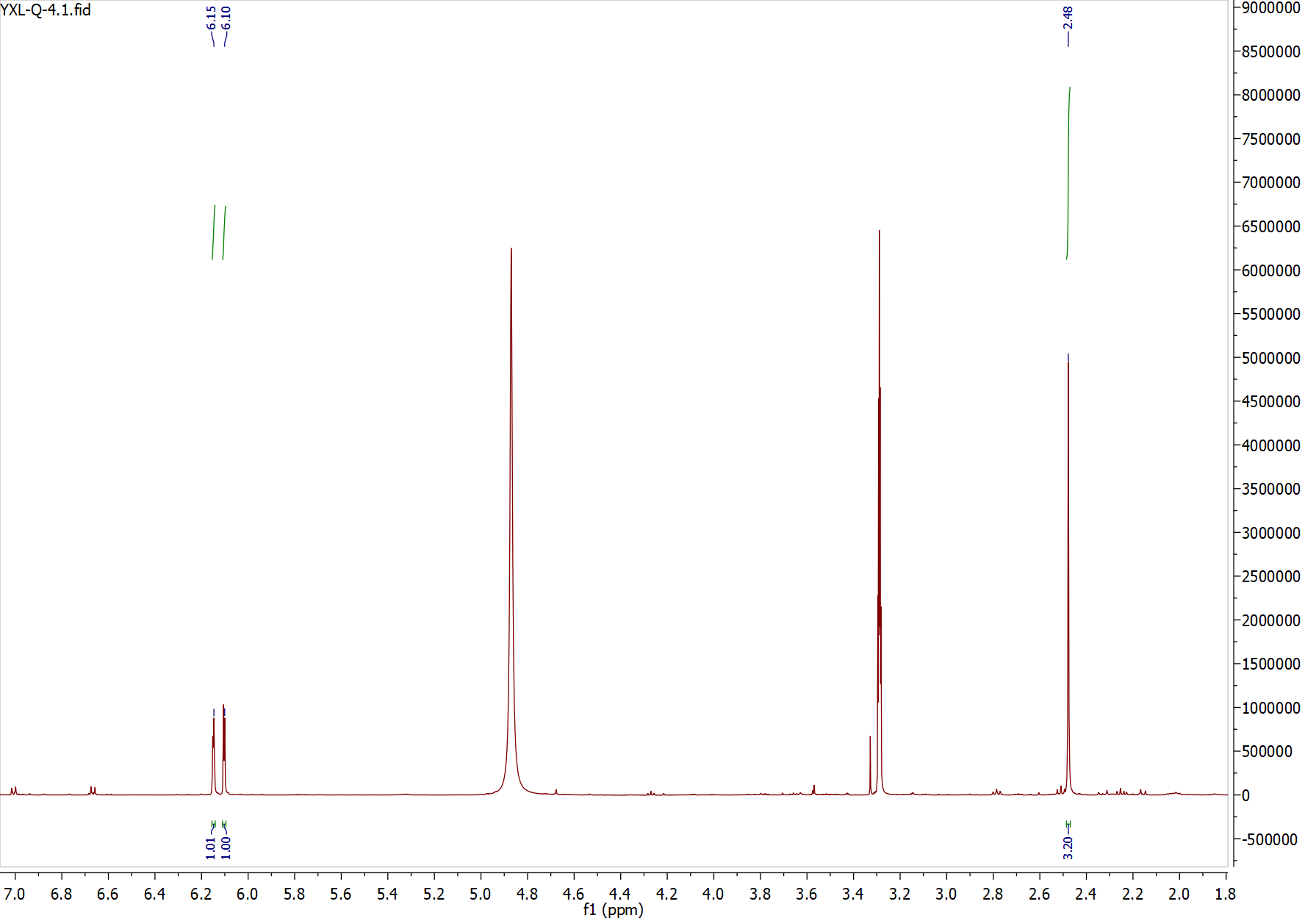


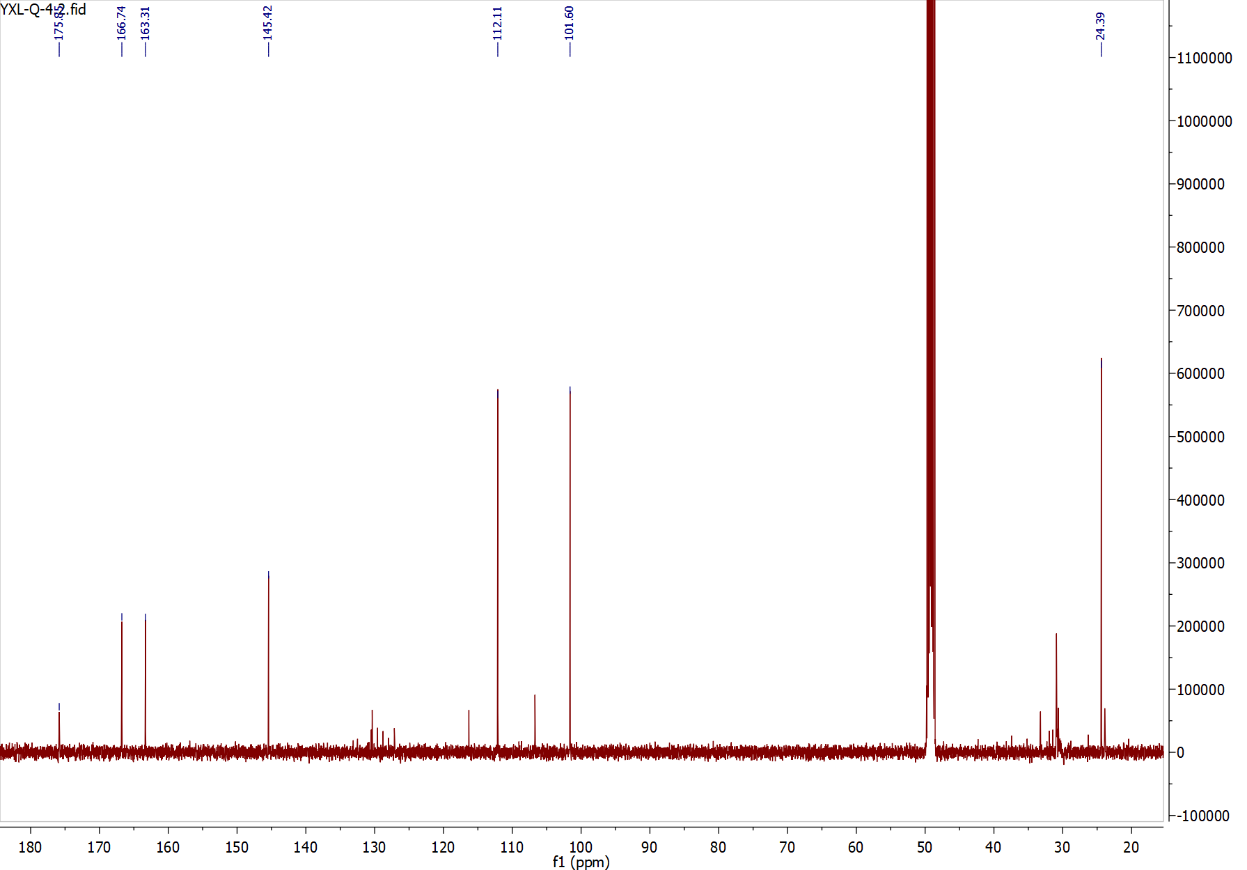


#

Figure S8. The HRESIMS spectra of compound 2 (lecanoric acid)

[M+H]+

[M-H]-

# Table S4. ^1^H (500 MHz) and ^13^C (125 MHz) NMR data for 2 in methanol-*d_4_* (*δ* in ppm, *J* in Hz)

| No. | δC | δH (m, J_HH_, area) |
| --- | --- | --- |
| 1 | 170.9, C |  |
| 2 | 105.4, C |  |
| 3 | 144.9, C |  |
| 4 | 113.0, CH | 6.27 (d, *J* = 3.1, 1H) |
| 5 | 166.7, C |  |
| 6 | 102.0, CH | 6.20 (d, *J* = 2.4, 1H) |
| 7 | 165.1, C |  |
| 8 | 24.4, CH_3_ | 2.54 (s, 3H) |
| 1’ | 174.5, C |  |
| 2’ | 117.3, C |  |
| 3’ | 144.8, C |  |
| 4’ | 113.1, CH | 6.63 (d, *J* = 2.0, 1H) |
| 5’ | 155.2, C |  |
| 6’ | 109.4, CH | 6.60 (d, *J* = 2.3, 1H) |
| 7’ | 164.8, C |  |
| 8’ | 23.8, CH_3_ | 2.56 (s, 3H) |

# Figure S9. The ^1^H (500 MHz), ^13^C-NMR (125 MHz) spectra of compound 2 in methanol-*d_4_*


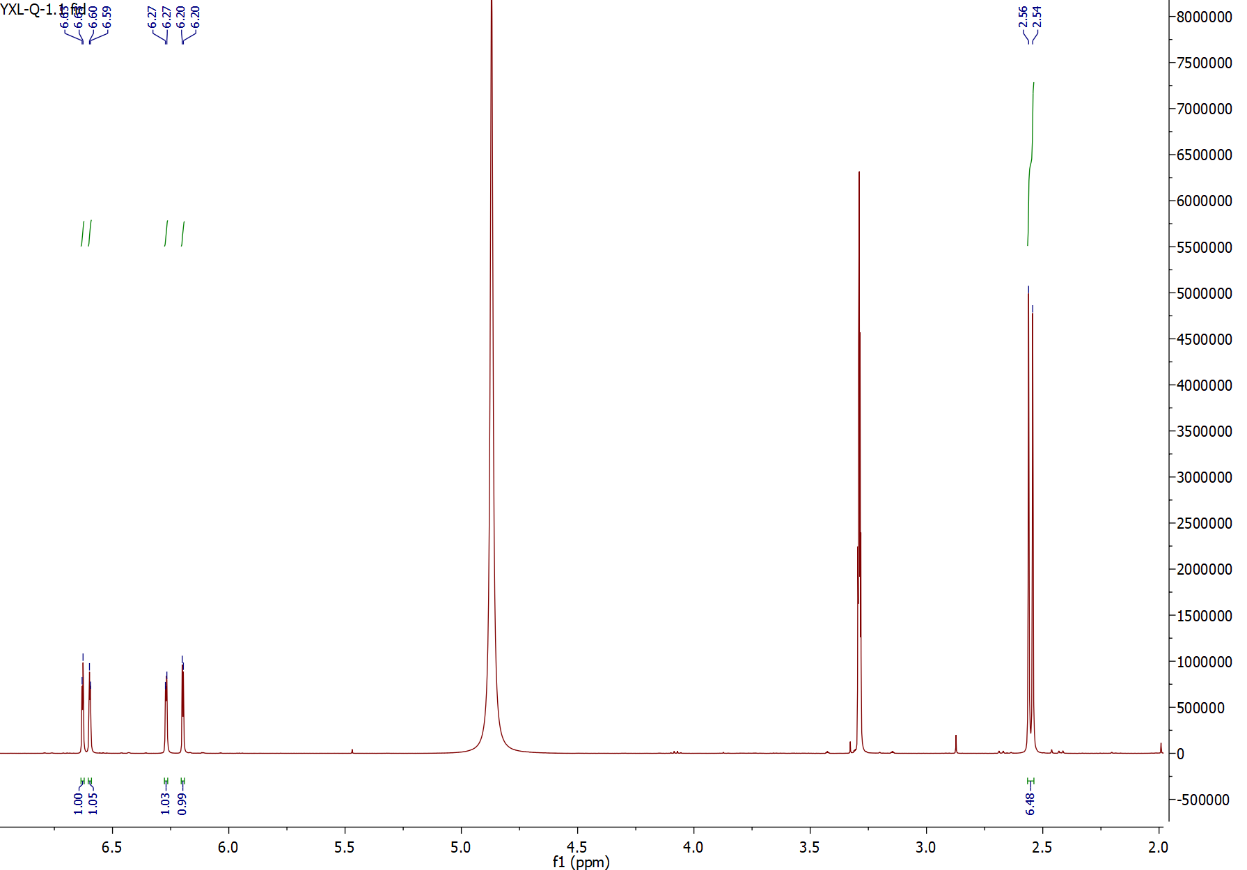


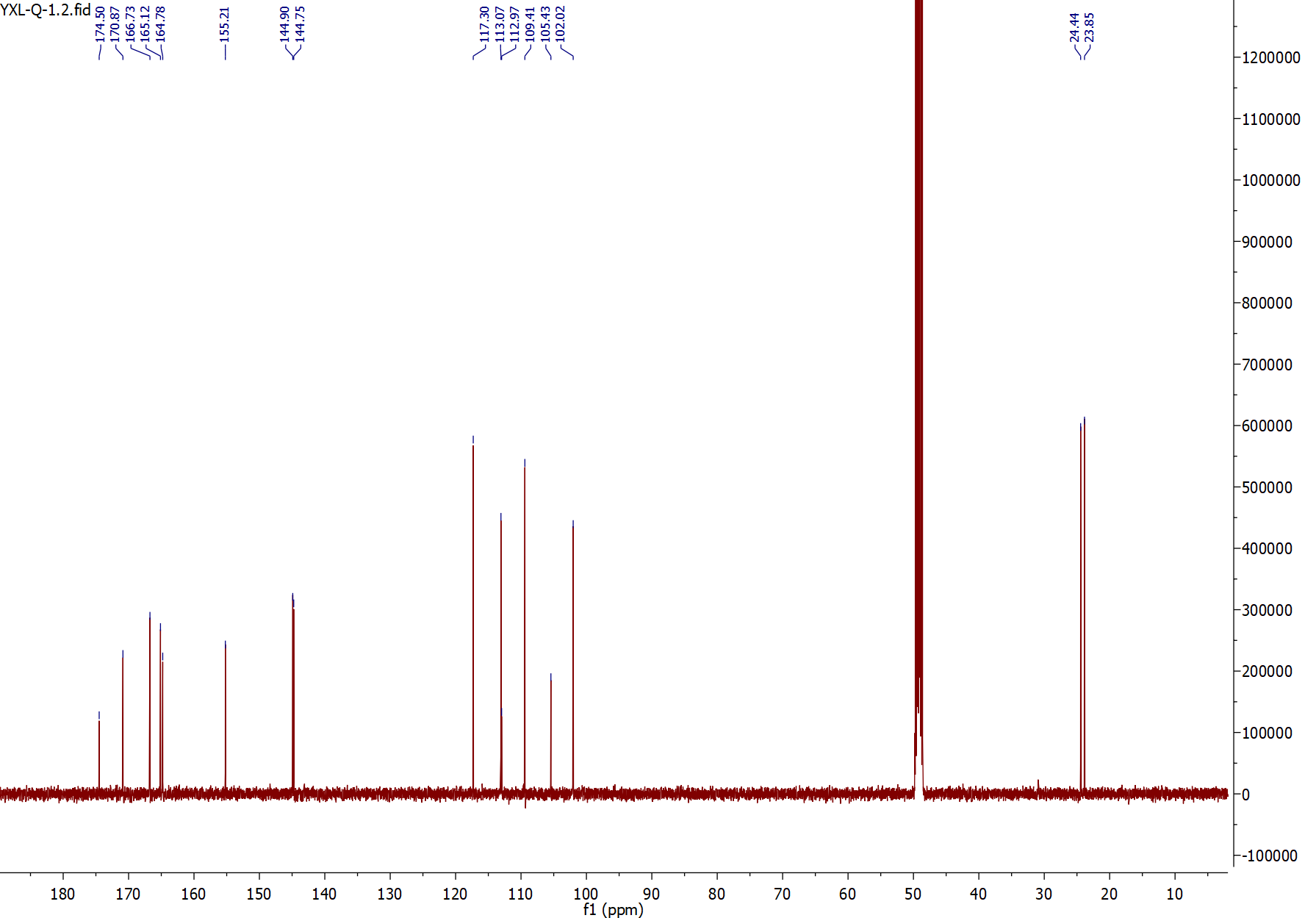


# Figure S10. The HRESIMS spectra of compound 3 (3-methylorsellinic acid)





[M+H]+

[M-H]-

# Table S5. ^1^H (500 MHz) and ^13^C (125 MHz) NMR data for 3 in methanol-*d_4_* (*δ* in ppm)

| No. | δC | δH (m, area) |
| --- | --- | --- |
| 1 | 111.4, C |  |
| 2 | 163.3, C |  |
| 3 | 109.0, C |  |
| 4 | 158.6, C |  |
| 5 | 110.1, CH | 6.10 (s, 1H) |
| 6 | 141.0, C |  |
| 7 | 177.6, C |  |
| 8 | 8.4, CH_3_ | 1.99 (s, 3H) |
| 9 | 23.8, CH_3_ | 2.50 (s, 3H) |

# Figure S11. The ^1^H (500 MHz), ^13^C-NMR (125 MHz) spectra of compound 3 in methanol-*d_4_*


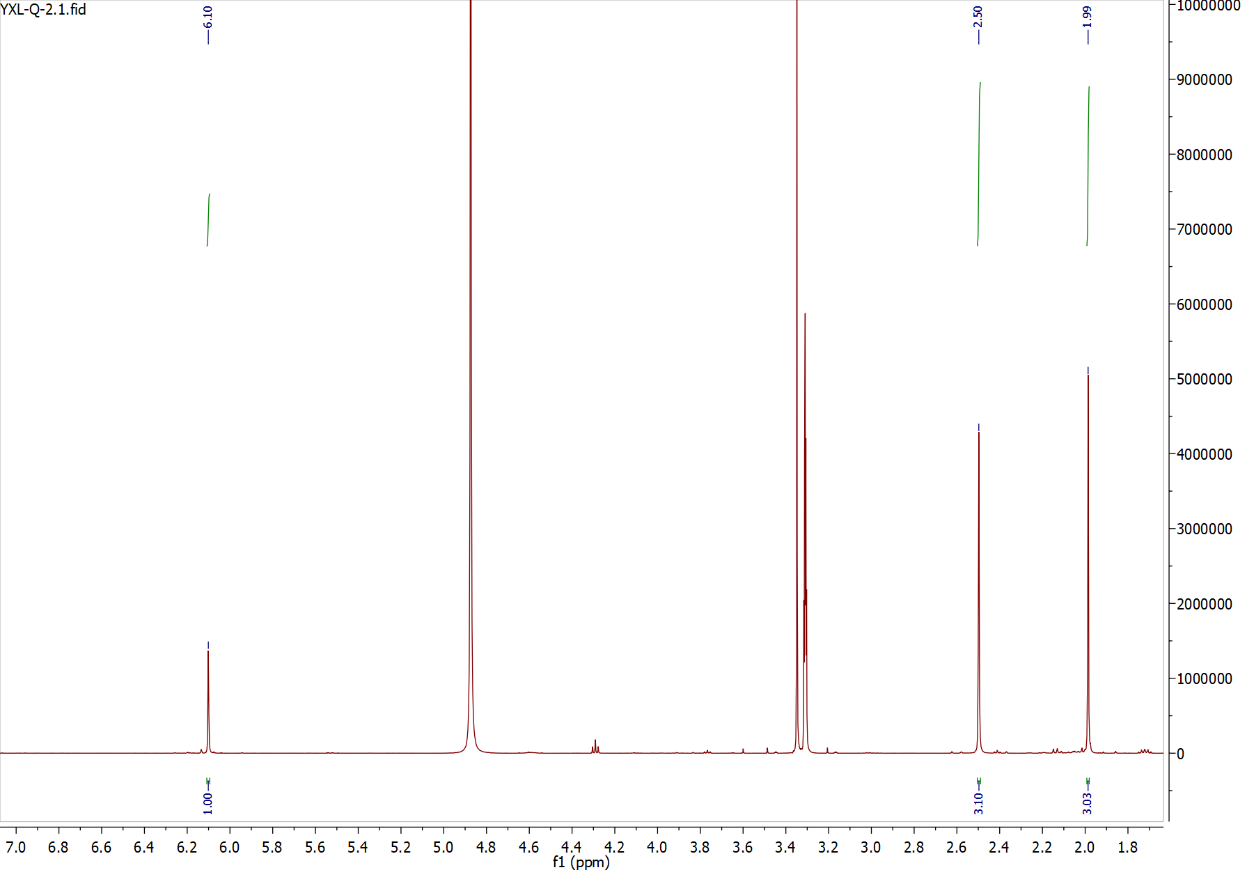


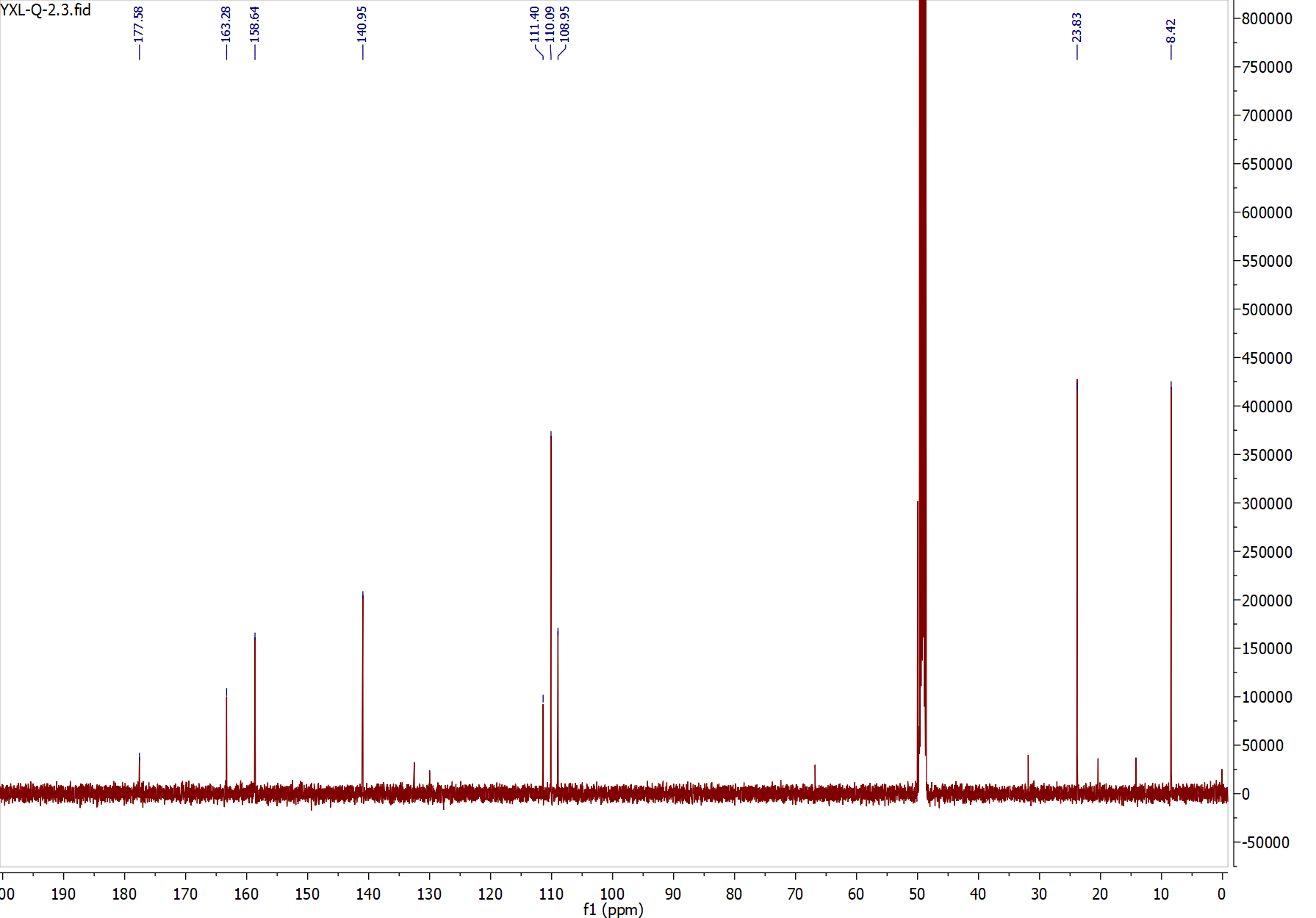


# Figure S12. HPLC traces (DAD, 300 nm) of crude extracts of Preu4





#

Figure S13. The HRESIMS spectra of compound 5

[M+H]+

[M-H]-

# Figure S14. The HRESIMS spectra of compound 6

[M+H]+

[M-H]-

# Figure S15. The ^1^H (500 MHz), ^13^C-NMR (125 MHz) spectra of compound 4 in chloroform-*d_4_*




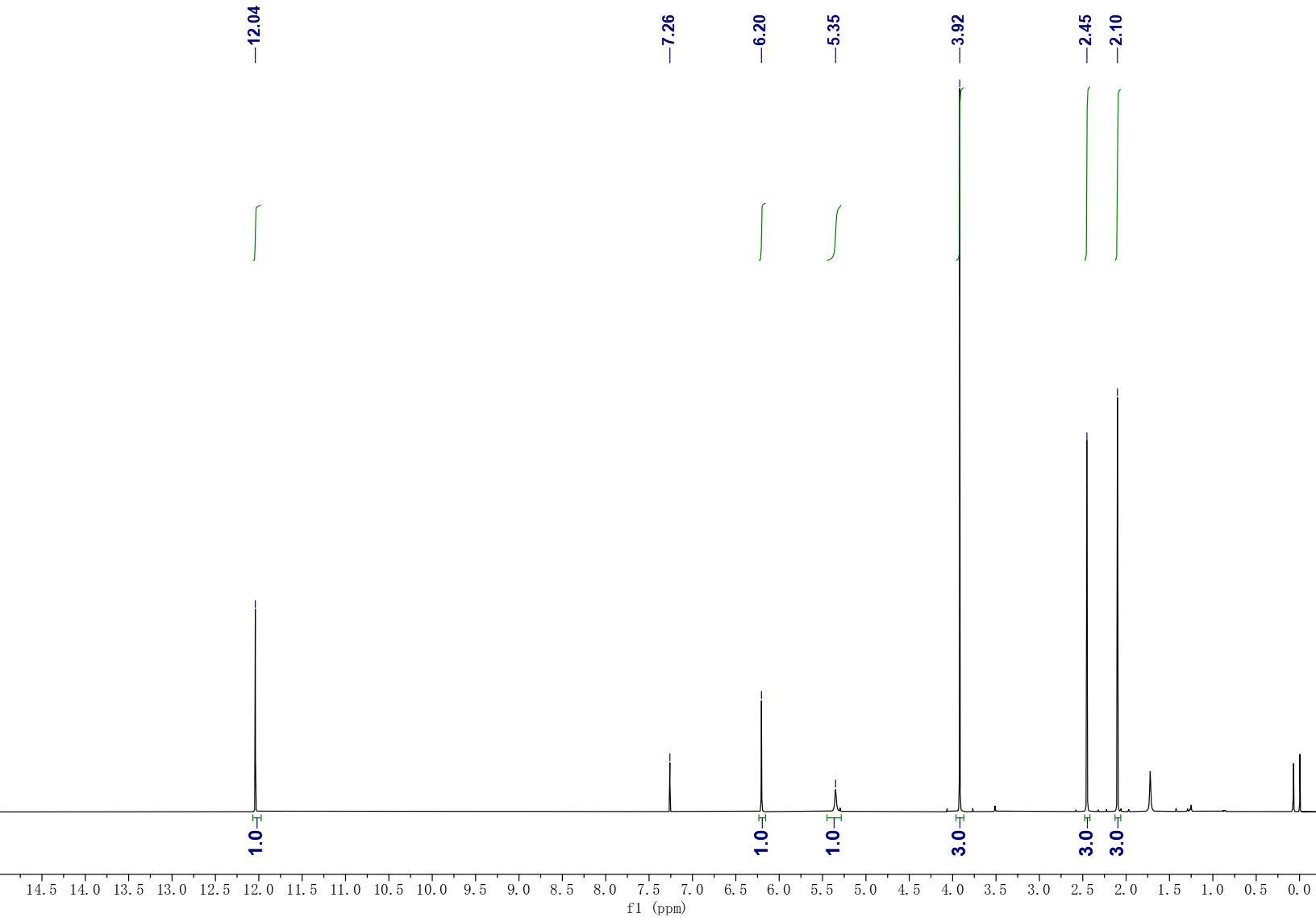


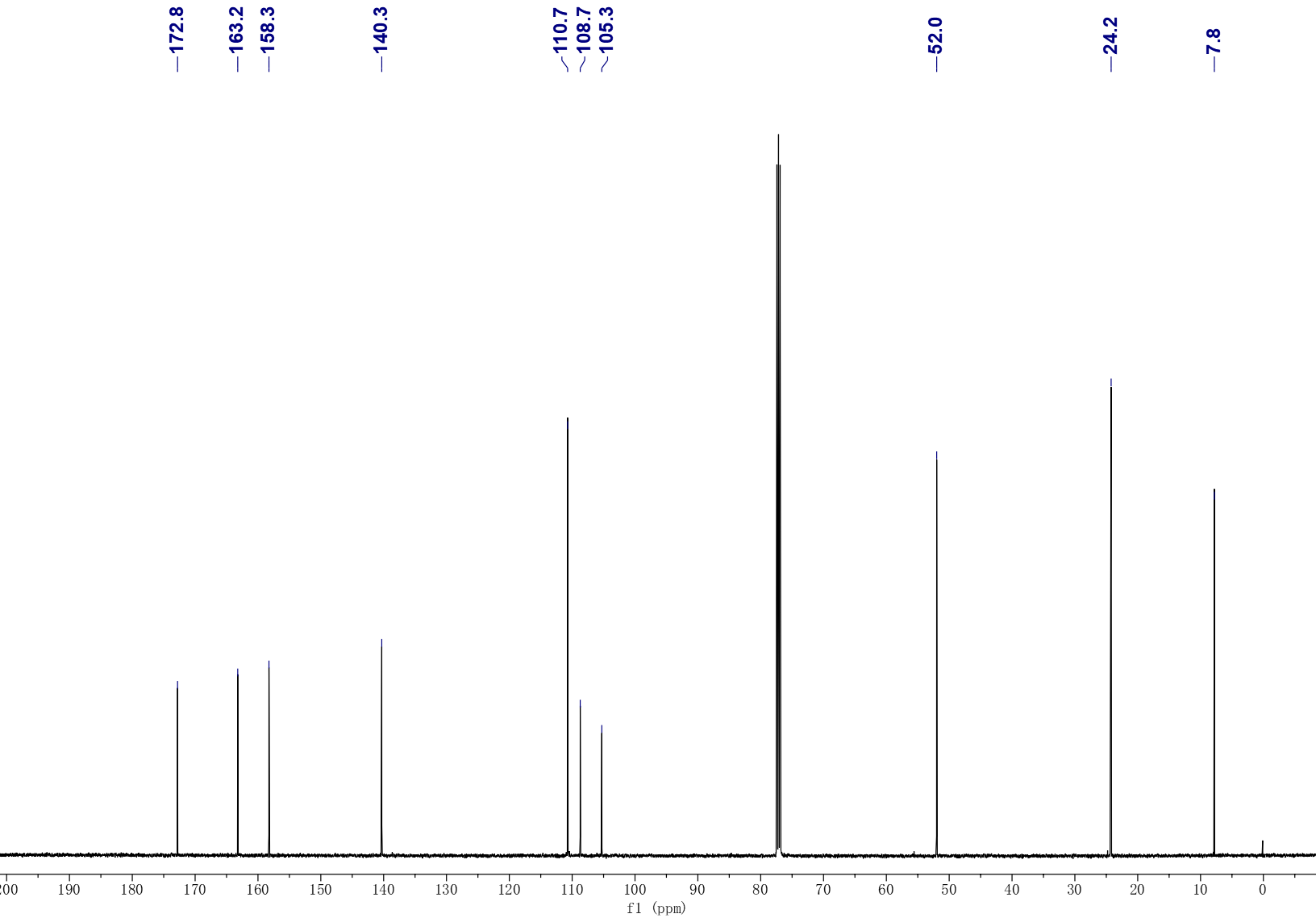


# Figure S16. Proposed mechanism of Preu3





# Figure S17. Proposed mechanism of Preu6





# Table S6. Sequence information for the fungal nrPKSs

| **GenBank accession** | **Protein name** | **Organism** | **Product type** | **Reference** |
| --- | --- | --- | --- | --- |
| AAD31436.3 | WdPKS1 | *Exophiala rmatitidis* | ATHN | Wheeler *et al*., 2008 |
| ABU63483.1 | PKS1 | *Elsinoe fawcettii* |  | Chung and Liao, 2008 |
| XP_756095.1 | Alb1 | *Aspergillus fumigatus* Af293 | Naphthopyrones | Perez *et al*., 2020 |
| AAU10633.1 | PKS12 | *Gibberella zeae* |  | Malz *et al*., 2005 |
| CAB92399.1 | PKS4 | *Gibberella fujikuroi* |  | Kim *et al*., 2005 |
| CAA46695.2 | WA | *Aspergillus nidulans* |  | Mayorga and Timberlake, 1992 |
| EDP55264.1 | PksP | *Aspergillus fumigatus* A1163 |  | Fedorova *et al*., 2008 |
| Q12053.1 | PKSA | *Aspergillus parasiticus* SU-1 | Anthraquinones | Korman *et al*., 2010 |
| BAE71314.1 | PKSA | *Aspergillus oryzae* |  | Tominaga *et al*., 2006 |
| AAS90093.1 | PksA | *Aspergillus flavus* |  | Ehrlich *et al*., 2004 |
| ACH72912.1 | AflC | *Aspergillus ochraceoroseus* |  | Ehrlich *et al*., 2010 |
| AAA81586.1 | PksST | *Aspergillus nidulans* |  | Yu and Leonard, 1995 |
| AAZ95017.1 | PKSA | *Dothistroma septosporum* |  | Zhang *et al*., 2007 |
| AAS66004.1 | AflC | *Aspergillus parasiticus* |  | Yu *et al*., 2004 |
| VBB75071.1 | PaPKS1 | *Podospora comata* | Isocoumarins | Coppin and Silar, 2007 |
| XP_001219763.1 |  | *Chaetomium globosum* CBS 148.51 |  | Cai *et al*., 2021 |
| ACD39770.1 | CcRadS2 | *Pochonia chlamydosporia* | RALs | Wang *et al*., 2008 |
| B3FWT6.1 | Rdc1 | *Pochonia chlamydosporia* |  | Winssinger *et al*., 2009 |
| C5H882.1 | Rads2 | *Floropilus chiversii* |  | Wang *et al*., 2008 |
| B3FWS8.1 | Hmp3 | *Hypomyces subiculosus* |  | Zhou *et al*., 2010 |
| ABB90282.1 | PKS13 | *Fusarium graminearum* |  | Kim *et al*., 2005 |
| AGC95321.1 | AtCURS2 | *Aspergillus terreus* | DALs | Xu *et al*., 2013 |
| A0A0N7D745.1 | Dhc5 | *Alternaria cinerariae* |  | Cochrane *et al*., 2015 |
| ASU91366.1 | PKS1 | *Nephromopsis pallescens* |  | Cochrane *et al*., 2015 |
| I3ZNU9.1 | ArmB | *Armillaria mellea* | OA esters | Lackner *et al*., 2013 |
| APH07628.1 | PKS2 | *Agaricomycetes* sp. |  | Braesel *et al*., 2017 |
| XP_007307184.1 | PKS1 | *Stereum hirsutum* FP-91666 SS1 |  | --- |
| EAA58470.1 | PkbA | *Aspergillus nidulans* FGSC A4 | 3-MOA | Sanchez *et al*., 2012 |
| F1DBA9.1 | MpaC | *Penicillium brevicompactum* | 5-MOA | Regueira *et al*., 2011 |
| EAQ84779.1 | CgsA | *Chaetomium globosum* CBS 148.51 | DMOA | Tsunematsu *et al*., 2012 |
| A0A097ZPE0.1 | AndM | *Aspergillus stellatus* |  | Matsuda *et al*., 2014 |
| B6HV32.2 | AdrD | *Penicillium rubens Wisconsin* 54-1255 |  | Matsuda *et al*., 2013 |
| A0A1E1FFN8.1 | PrhL | *Penicillium brasilianum* |  | Matsuda *et al*., 2016 |
| Q0C8A4.2 | Trt4 | *Aspergillus terreus* NIH2624 |  | Itoh *et al*., 2012 |
| Q5ATJ7.1 | AusA | *Aspergillus nidulans* FGSC A4 |  | Yeh *et al*., 2013 |
| Q5AUX1.1 | OrsA | *Aspergillus nidulans* FGSC A4 | OA | Gressler *et al*., 2015 |
| CCE33500.1 | PKS7 | *Claviceps purpurea* 20.1 |  | Lünne *et al*., 2020 |

# Amino acid sequences of polyketide synthases Preu1-Preu9

**Ⅰ Preu1**

MASGPRNDDIAIVGLACRFPGGASNETKLWDLLSNRESAFTEVPPERFNVDAYHHPSPNKLNTLNSRGAHFMHEDVSAFDAPFFGITAQEANAMDPAARMLLELTFEALESAGQKLEDVAGSDTSCYVGCFTRDYHEMLYRDIESAPMYTGTGTGFSLFSNRISWFYDFRGPSMTLDTACSSSLVGLHLACRGLQAGESKMAVVCGANVILSPDIALTLSNLHMLSMDGLSRSFAEGTTGYGRGEGIASLILKRVEDALRDGDPIRAVIRGSGVNQDGHTKGITVPSSEAQADLIQTVYSSAGLDPSETGYFEAHGTGTAVGDPLELGAIAKSISKSRKVENKLVVGSIKSNIGHLEGAAGLAGVIKSVLMVEKNAILPNIHFEKPNRRIPFEQWKIKIPTELMPWPASGVRRASVNSFGYGGTNAHVILDDAESYLQKRLSNGHSEENGMELLKETRIFLLSARDEASLERLKQVYSDYIADVAGRRSPDNLFDESAYLDGLAYTLGCRRSVFPCRSFITASTLSELQESLSVQRLISTKAATSSRLGFVFTGQGAQWARMGLVLMDYPVFAQSIQEADRYLSEELRSSWSVLEELGKDAEHSQIGLAEFSQPLCTILQVALVDLLTSWQILPATVVGHSSGEIAAAYSFGAITKQDAWKISYWRGQLCAQLPLKRPELRGAMMAVGLGREEALSYIEKTGNGQAVIACVNSPSSVTLSGDETAINDIESALQAQNIFARKLNVQNAYHSHHMQPLADEYLAALEGLSTLSQEKAGTVKMASSVTGALINHTDLGPSYWVQNLVSPVLFSNAVEALLKQSTKGRRQARANEPAFDYLVEIGPHAALKGPLRQILQAHEASQIPYSSVLMRGEDGPVAALSAAGDLVCRGIQVDIKAVNRFQGRAIPLTNLPTYPWNHSLKYWADSRVSRARQHRKYGRHDLLGAPTQDSDELEPRWRHFLRVSDNPWIQDHIVHSSILYPGSGILAMPLHALQTLADSHRDVESIGLRDVSIVKAIVVPDDQFGLEVFLRMRRQRPRNGTWNGWWEFSVCSNQENDHVEEHGFGLGKIHYRPEKDTSVKTATNHVNDEFEREFDEVQATSTASISPVDFYAVAKSVGLTYGPSFQGLTEINAGNAKCSWKIQVPDTQKIMPAGVESPHIVHPTTLDIVFHSLFAAIGDGHLDMQHAAVPIGLKSMKISVDLPTGGNTFLKGISKVSRDAGRDIVADIRVAAESSNTPSIIVEGLRCRELPNGNSQSGPSETIKAPIGHVVQKIDVDLIEPIQLAQHIQKRFLERKEDGTLASEDLGEAIITIVDLVAHKNPRSSLLQVGGVTPELTQRILSNLEADNVSAKRFKNIKVVDANQELISQLETQYATPAGTVQFEKVTLDEAQSLAELKESSIDLAIVNIEELHSDKVSEYLANVLRSLKPGGKVLKIDSAGRNGAIGASDTLSFDTLCAGPEHVLSFATKGTAEKANTENFRTVILLPRCPSENVKKVASALEQVITVKGAIDTVIWSAEMPSLENSSTVISLLEFDDAFVADLSEEDFSSLKTLALGVKRILWVAHGTDPQMQTAAGWLRSLSNENMGIDYCYLLLESATDQEALDVAKLIERVSSTEEMEREYTERDDGLCCSRWAAKTELSALVGADSDQSKDATMRLGEARHGLTLTGKRGKLPSDARFTSVDVLDQNLTENEVMIDVRSVLLSTNDVQGTGQTGWREAAGVVVATGTAGSFEVGTAVSFVYDGPISTKAKINSKYCSTLSGTPEFQDALLQSVTYPTVYHGLCTLGQLRCDKTIFVQGGSSILGQAAISLARRLGATVFASVRDEEQNSILQRLGVPSDKILNDNVCERSSIIKRVNEGRGFDVIFNATGDEETIAELWQCIARGGKFINADANKKDTPATFNLSAKPFTMGASFEIIDMNEYLTNDFSTYQSIRDESESFRTRCSAVGLQIPVFTAGNIHEALDSVHVPTGLGKAVLLFSPDSKIPVSPDVKNQLRLRPDATYVLAGGLGGLGRSLAKLMVGSGARHLAFLSRSGPGSAAAQSISEEFSPLGVTVNFYGCDVADSESVSHTFGNIAKNQALPPIRGIIQSAAVLRDSIFENMSHTQWTEAVRPKVQGSWNLHQASLSGPCAKEGLDFFVMLASISGFVGNRGQANYAGGNSFQDALAKYRKSLGLAATSVDLGLMQDIGLIAERGGQSNLSDDTVVPLTAKDFELIFKLAMNSEGHDVPAQIVTGLPTGGILQKQGIETLPFYYRDPRFSAMQFMDLDETLTSAGGSAGNESVSMEEQLASAKSREQANGIVLEALRAQVAKALRCPAEDIDTARPLHYYGMDSLMAVDMRGWVQGKLKAEISLFDVMSGSSISALAEKISKASKLIKAELE

**Ⅱ Preu2**

MMAVHLAVASLRRNESTIAFACGTHLNLEPNDWVSLTKMNMISPDGRSKMFDELANGYGRGEGIGVICLKRLDAAIRDGDHIECVIRETGTNQDGHTKGITAPSAEAQAALIRKTYRSAGLDPTQVGSRPSFFEAHGTGTHVGDPLEAKAIQAAFFPPGQEYSDDEVLYIGSVKTIVGHTEGTAGIAGLLRAALAVRYGSIPPNLLYTRMNAEVAQYANHLRVVTAANPWPTLPVGCPRRASVNSFGFGGSNVHVIVESYVNTAARRVTSIPSFGAREPIFTPFTFSAISERALMSLMEKYLKYLEDPKSSVSPRDLAWTMQYKRSEFPFRSAMSALTVEDLRKQLRKATDILSDESNTSHIIRASQSTQPLIMAIFTGQGAQWPGMGKDIIEQSPHACDIIARLDDSLASLPEDDRPTWKIREELAIDSSRSRMDEAALSQPLTTAVQILLVDLLDIAGVKVRTVVGHSSGEIAAAYAAGMITAEDAIRVAYYRGLHSKLAGGVAGQKGAMITTFLTPNQAVDLCNLPEFRGRIVPAAFNGPTAVTLSGDVDAVERVLLMLEAQNIFARKLNVEKAYHSHHMKACAPPYIQSLEQCGVPVMPPFMDSPIWFSSVNPGKRMDPQKALHGSYWAENLLSPVRFSDAITTAISETGVPDIFLEIGPHPALMKPVLQIISDVAKDGTVYSGLLKRSSNSILSFSNAMGSIWERFGRCAVNFSKLETTLSGGSPPVPAKDLPTYPWLHDREYWWENRWLRRRFEAAYPPNELLGEELSMGAQHETKWRSFLQPKDVPWLLDHKLNGVAVLPGAAYVAMAATAARRIYRKQTIIMIELNDLSFKLPITFPDDHTSIETVLTVVNIRSTTQQGQADFVFDFCSHQRQDELMTAARGSLTVQFGDDVNRTYPERLSQHSALTELDLDTFYNNLEGRGYNYTGPFRSITSLQRRMNFSTGRMDFTPSDMTFHPALLDGLFQATFAAENYPGDSAMLDFRVPSLVRSIKVFPARCDEMTAMMKEKVDFQVSRTGPSEYSGLLLCENGSGTAIQMDGFCTAPFRMSTPEEDVKMFSEVAWRQYVRDARSLTSICVLAAHEKEPTLACERVGFYYLRRLNETIPPEEEKQAAPHLRRLLEFARMVVCENLLGLLSHMSPEWIHDTEADIAKIIAEYSKLIDMRLVDAVGRAYPSIIRGHVSALSVLTEDGMLTQLYSEGLGFYQANSTMTRLVSTISNQYPNIHILEVGAGTGSATSGILPTTSYSSYTFTDVSPAFLATAKDKFRRYSDKMAFTTLDLDKDFEDQGFTANSFEVIVASNVLHAVTDINASLIRIRRLLRPGGYLVCTELPESYCVKTTVIMGALPGWWQGAPRGRNWSPALTEPQWDRVLKDTGFAGLDAISPLDNDLSQSYRVFVAQAVDERVVSLRDPLTHPPSRSHDSIMIIGCESLVKSQFLEQAGQILLPSFQDVIHVPRLEEITQDVTVPYSVLCLAELGKPVFQDMTAAKWSALQTLLGQATDIIWVTTGHKSPKKVEESYKSMAIGLGRVVRNELRDLRLRFVDVDDLNTLTARSVSQATLEWYMLGQWAAQGWGNQVLFPHDTELAFENGLILAPSVVHSKTKNDHYNSQQRRIVREAQPYRVPIELVYSLSTKQYDLHEAHHVPAYALEDTITMKILYTTLYALKLKKVGFLFIGMGICPDGKYALVVSEKNGSILRLPRHTVYPFTSSRKPTQRDLWITAARIIAGRVVSGCNLTGKLLVLVTDATLLTIIRDEATRQHKSVVFITSNPNFASKQALFLHHSALDVQVRQSIPSQIGLLVNLSNRLDDKNLFKRVRSVLRDTCTEIKTIDAILRTTSSRYWSPETLGEIKVDVVDEKYLMETANMPGNMSNQAEPTILSPKNVSKQAFNNPLTVLDWTVTTHIPVTIQPATAIAKFSGNKCYIIIGTSDLAQSVCELMVSNGAKYVVMASRNPTRLSGWVQDMASRGAYIDIKSVDVTDAMSVRKMFSSIRNLTNDRGTSAPPIAGLVHMGLGLKDAAFSALTFEDLQIATDVKAKGSLLLHEQLQEEKLDFFILTSSISYVAGNPGQANYSCANAFMAGLANYRRDMGLAASVVHLGHVAGVGYITRMSQARGVVMEDARKHGLLPISERDLHQIYAEAVLASPADSGRNPEIITGFPELTSDMLESSVWGKQPIFTHLVTAGTRPSTIVQPKPHLSVRERLNNQLSSTITSAPEKAESSSHDIIRNGLVERLSVLLQVDVKQIDEDISLLDMGIDSLVASEIGSWARKELRVQIPHSMIFGGASVANIVDFAVAHLDKEWLALKNGSGEGVGKGK

**Ⅲ Preu3**

MNPPSALAFGPEERIPTASNLRLLKDVLQDDPTFAGITACLKQLPDTWKALLHQDAQLQSLASERRAAVLSNSLLNDEQHEGDMDTNQVIMPMTVLVHMVQYRQFLQQSSPSSHATVMQSVAAGGVQGFCAGLLSAFAVCSMTNEDDFDACATYAIKLAMCVGAYVDLAMESEKGDMASAIVRWSIPDGRNRVNKAVGRYQSAYISAISDEDNVTVTASRPDLDAICTSLGSTGMSSKILAMTGSFHHPKNFDLLQRMISLLRAPQLAPSTKFTNALLRSNSTGELLTGAKTIENILEDILCKTADWRLTMANTSKALRSGNGSRPNIHTFGLVEFIPSFVKNEFNILTQRLAPTAKEQTGASPSKSTLQYNDNAVAVVGMACRFPGADDLDEFWELLQSGKSMHERMPADRFSTTGLRRSNDGAPFWGNFLKDIDAFDHQFFKKSSREAAAMDPQQRLLLQCAYVAMENAGYFDPSVQHKIRDTGVYLGACSSDYNDNVASHKPTAYSTLGTLRAFLTGRISHYFDWTGPSVVYDTACSSSAVAIDAACKAILAGDCQQALAGGVSLYTSPNFYQNLDAASFLSQTGPCKPFDANADGYCRGEGVGLVVLKKLSDAIRCGDKIVAVIASTGVNQNRNCTGITVPHGGSQADLYRRVVAKSGLNASQVSYVEAHGTGTPVGDPIEFTSIKSVFANPDITRDEPLTIASVKGSIGHLEGAAGVASLIKVCLMLQHSAIPPQANFTKPNPNLGGVDMRNIVIPTSSIPWKARNKVACINNYGAAGSNGAMIVCQPSEPASKTQTRLPSQSLSYPLFISGDGTDAVEANCRAIAKYARQLQQKRAPSVVASLAYRLATSQNQNLSYAMVTTISENGDIESTLTKASATLTQPRSKAKQSVVLCFGGQVKAFVGLDQQLFDSSSILQKHLRLCDSTMRDLGYPSIFPAIFQSEPLKDPVQLHGVLFAMQYSSAKAWLDCSLQVDAVVGHSFGQLTALTVAGVLSLKDGLRLVCGRAHLIKTKWGSATGAMIAIEAPLIRVQEILSKISVAGHEAEIACYNARESHVLVGTTTAIDAVRTFVLESGIKHKRLPVTHGFHSTFTEALLPGLRELAKGLQFKSPIIPIETCTEYKSWEQATADMITKHTREPVYFVHAIERLSARLGPCTWVEAGTGASTPAMIKRCLPDSCADSFIHATLESNKAFGSLADATANLWRCSQPVQFWPFHASDRGRYVPLNLPGYQFRKTKHWLEWQDTVALPAFLEKEPSTSEPKGHELLTFSSFEDTSKSVAAFKVDPESDEFMMLVKGHAVVAQPLCPAPLYCELALRAIKHLSPETASNAPDIRDLQIHAPLGLKTNRNIRLVVQKNSIPGHWTFTVKSSMGSDDELTHAHGLVAFGGTVEQELASYQRLIGHQKIQSLMTDPECDALRGSATYKAFNRVVTYSSYYKGVQAIYGRQNEACGKIELSSGEEQMAQARGILTPLLADNFIQIAGLQINVLGDCEDHLVFVCTETQRIIYGPGLHQQPAARYEVYSTISQNGPKEVMSDVVVFDPATKNVEFVALGCRFTRVTVPGLRNALQAANGDARAQERPSGSRISPSPLAPELPAKIQIQSRENLDITEKSGRGKPPRVENIQIATPKVDYLAQVKALLHKVSDVPIDTIQKDSTLDDLGIDSLMVMEVQTEVHSEFQLTIPNKDWATLETPGKLAEYLAKTLGGSVPDSAPPGVQRVPALVISDAEQSSDESPYDSTDDSASGYGDLDIDTAATTPGIFATRDSSPFRKAALDSPNPVNKVAQRTFSDIRPKYDVFAAEEGFAGFWRDVYPRQKRLTLAYVVEAFAVMGCDLSDLAAGQILPKIEYLPQHVSLIKQLYVILADSTLITIENGTYRRTRVSVDTTPASDLLADILRAFPQHAEEHRLLDVTGSRLGDCLIGRADPLRLLFMDRANKELLDSVYANGPMYKAMSRLLGSYILDTMQQWQGQKPLRILEIGGGTGGTTKHIVKLLHQQGIDFTYCFSDLSRALVTKAKKTFSIYPQMEYMVLDIEAPPSSEYLGQFDLILSTNCIHATKNIQQTTKHMRQLLSSEGFICLVEFTRNIFWFDLVFGLLDGWWLFEDGRPHVLADENLWDQSLRAAGYGDVQWTEGQSEESKTLRLIAAFNVSNEDAKAANALASALAVPGRKGRTSATTIRWKQEGDLDLMADVYLPSDLDASTVSRPVALILHGGGHVLHTRKHINPRHIKMLQDLGFLPVSVDYRLCPEVNIRDGPMTDACEAVDWARNILPCLPVCSELRVDKEHVVVIGYSTGGHLALTTAFTTRVRGFKPPSAILGFYCPTNYSADWWRSPIYPELAQQSSSETFDLLEGVNEHAIAGYTPTVNNNVAALLMSLDDPRWRFVLHANWRAQTLPMLINGLPSKSRLARSGQTVDSVINREIPDAEDVASISPYDQIVRGSYSTPTFLLHGTKDDLIPWQQSIATVDALARRGVNARVEIIEGAEHCFDVWSDKYDGMIGRALEWLVEQCRNA

**Ⅳ Preu4**

MEAVVSSAPFIAPIDSFEHCQIFLFGDLASPFEDDLAQLLHCKNNALLQSFFDQVNRAYRTEFATLPAEQQEWLPRFTDLVDLVSNLNSTIGARALRFGLLCVYQLGRLIHSDVLPHVDTSYLIGVCTGSFAAAAVSASRTMAELVPAGVEAALAAFRTGLHSFKAQQDIVPTTVEQLQSWSFVTSMTESQALETIREFTDKMHLTQSQRPYLSAVSPSSVTISGPPRTLKHLIESCAIKAHPIQVQSPYHAAHLNGPDEVDDVLGHLRDERLSEYRQHIPIWSIATGRDVPAHDFHGLLRRAIDETLCEQVRWDKLCPALKSHLAQRSSLKSCILFPVTSNAATLLSSALQHESRLDVTISNALNTRVERVNTSAAPGKFSDSKIAIVGFSGRYPEAASNDELWEVLKSARDCHRTIPEDRFDWAAHYDAAGKKKNHTRVKFGCFINDPGVFDARFFNLSPREAENTDPAQRLALMSAYEAIEMAGLVPDRTPSTQRDRIGVFFGVTSDDWREVNSGQDIDTYFIPGGNRAFVPGRISYFFRFSGPSISVDTACSSSFAAISTACSYLWQGDCDSAIAGGTNVLTNPDNFVGLDRGHFLSQTGNCNPFDDSASGYCRADAVGAVVLKRLEDALADHDPIFGVIAGCSTNHCGQTVSITRPHEGDQLALFKRILRHSNTDPRDVGYVEMHGTGTQAGDAAEMRSVLAAFANDHLRPRPLHLGAIKANVGHSESASGVTALIKVLMMMQHSEIPPHRLNGKLNRNYPSDLADRNVHIPFKTTPWRREDAGGKRMAFLNNFSAAGGNTAVLLEDAPVLEDGENVVSDPRTVLPLTVSAKTGKALTENIHALVNYLNDNPDTSLSALSYTTTARRIHHKFRVLVSGSDVAAVKAALQRREHDVHKIKTVPPTVAFVFTGQGTLYAAIGKEFFEHVAIFRTDLVRFDRIAQRHGFPSFLGLIDGGEEHVDPLKVDPVVAHLAVTCVQMALAHIWRLWVGEPSCVTGHSLGEYAALYTAGVLSASDTIYLVGSRARLLTKHCNKLTHGMLAVKAPAAVVAAELASSGSDCTIACINSPVSTTVSGPKKAVQDLSTWLKTRNIDAIPLEIPYAFHSAHVDPILHEFENLASSVHFHEPAVPYLSPLLSWTVTEPGILTASYLARSSRQCVNFLGAIEHARETKLVNDTTIWLELGTHPACSGMLRQIIGPQASTVPVLRKDGHIWKVVVAALDTLYHNGVEIQWNEFHRGLDAAQRVLALPSYKWTLQRYWIQYRHNFCLTKGDDPATLVQQTIPPPPLPARTPSLSSSVHRVLESNNSADSSTLLVESDLHDPRMAAVVSGHKVNTAMLCPSSLYADMALTIAKHMLKSNGMLHEKTGLDCRSMAIQRPLIVQPEATSQLLRVSAEAQWPKNEISLIFFSVSANGRKLAEHATCVVKVTQDQSWLTEWKRNAYLISSRIASLHKAVDTGGAHKLKRGLTYKLFASLVDYARDYQGMEQVILDSECLEATAEVKFQIDNQGFDWNPCWIDSLGHIAGFIMNGNDNICSQDQVFINHGWEAMRCGKAVEYGKTYTTYNRMQLEGGTMYVGDTYIFEGQQIVAVFEGVRFQGVNRSVLDHLLPGKTRAVTQQVASKPDASGHAGTGASAVLVKKRTSPALATKPAPITAKDQRSQLGKTSGIIKIILEEAQMDEDELDVDAEFADLGVDSLLSLTIASRLQDELGIEIPTSTFMDHPTIKAFISFIGGEESLGSSSAQSVASGEESYFTDATSVDGENVDSDTDIMTIIRTIISEETGTPLEDISSSSPLAELGLDSLLGLTIMGRLSEQVDVCLPTSLFVDSETLEVIEAALREAGLINKNGKRGYRAKSQDAASIKAMDTKPVTASQSSFDLNTPPHATSVLLQGSSRTAKKTLFLFPDGAGSATSYHALGNISSNVVVYGLNCPWLKTPADLKCSLEEYVAKFLIEVRRRQPTGPYHFGGASAGGILAYEAAQQLDRAGERVATLVLLDSPDPVGLENPNQRMYDFLDSMGMFGMSGKQAPQWLRPHFDAFLALLDAYEVKKFRGSSPPTTYIIYARDGMCKHESDPRPEVRPDDPREMLWLLNNRTDFSGAGWNSLVGKENLHVSVLGNVNHYTIMQAGPQMKELCARIARALQG

**Ⅴ Preu5**

MTTNDTPIAIIGLSYRAPGVGRKGLWEYLSQARSAWTTVPTDRFDHSAYYKAGADRSGVSRVRGAHFIDDVYGFDAAFFNMRAEEAKNSDPQHRLLLECALEAAEDAGHSLLSIAGKKIGVFVGSGQHEYSQRLGDDEFATHTFAGTGVAPCMAANRISYFFDIDGPSVVTDAACASSVYAAHVAVSALRNGDCEAAFIGSASLNIGPGGWIVLEKTGALSEHGRSYSYDEKASGFGRGEGAGCLLVKRLDDAIRDGDPIRALIRNSACNHSGRSEGITMPNGLAQQKLLWNVHNAVGLHPGETPVVEGHGTGTAAGDPIEAGAFTAVLGKDRTAENPLYLGSIKSNFGHLEGASGMLAMVKAIMMVENGIVLPTAGFEKINPKIKDAEKIKVAETPLPWPKGEKKRAIVTNFGFGGSNSAIVIEKAPSRDELGHETNGTNGVSVSNGVNGSNGFTNGSNGTNGHAENGNGISAQERRLYTFSAKTEKSLNAYLSSFDEYLDEAPEDSDFAKDLSYTLGQRRTQHPYRVSVVADSVEDLQEKLSTVKPSRIKDRVIAFVFTGQGAQYAQMASELGHFKVFASALKDADAQLKAIGASWSLVSELAKPAEESRVDVAEISQPACTAVQLALVELLKSWGITPTAVTGHSSGEIGAAYAAGLITFRTAIAVAYFRGQAAALLAHKQTSKGAMLALGVGFEEASALIKEHSGDAYATVAAINSPKSVTVSGDVAALEAIHKVAEEQGLFARRLKIEMAYHSRHMEAVADYYLKAIAPFCEKNESFVSKSNAARPIFVSSVTGHVEDNSVVDATYWVKNLLQPVRFADSISGLFTQLGEDKSKIPNVIVEVGPHAALKSPIKQTVESLQLQGKSAFTYLPSLVRNVDGDQALLDLAGSLFTMGAPIQLGGVNQTDSKNAQVITGLPAYEWDKSAHYELKPRPTHEKLFPGEEYHELLGRRVVSNGGKERAWRQVFTLDEMPWIRDHVVAGATIFPMTAYMSAAIEACRRTLPVSSPASAFLVQNFHVVRSMEIAEEESVELMTKLVPAATGEGTTSSTAWAFEISTYKEESGWTIHAYGQIEPEFADMSLETPTFKASLPLVDTTADLLEHDIEGTYASAGVRATRYGPTFRNNVRFWEGKGYTVLEHRLRDLGQALHEPVTRGSPVSVDPPTLDGFLQGGGPLQVDEDGRRPAQMPNYINRFRVSNKIPSDPQTRFDVVMRRLDYDVKGGRMHVGVAAFARNTDGTLSPIAEWESAAFRNIGSADENIDPASDVPDNWAWEKLPRYDFLSLEELRKTLSVGSLGEEEGIRSTNLEKAAVWYIGQALKKTVNDDFSELEDHLQRFLVWAKKTEAEYHTKFDEEPTELLQQVRDHDAQGALLCFIGEQLVPILRGEVEALEIMLAEGRLTKHYEADVVNAHLSQAVGDLADNLSNLEPSLKILEVGGGTAGTTLPILEALSRGRDEPGFLNYTFTDISAGFFEGSRQKLAKWQSRITFKKLDITKDPIDQGFSASDFDIVIAANVLHATPDMVQTMTNVRTLLKPGGKVFLLEANMHPPSVLPFSLLPGWWAAEDKYRDHAEGPMMPVKVWDQLLLDSGFSGVDVAIPGVWDSEVQLMSIMASTKIAQQEGKITICGASLDDKEIAFAKNVDDALSKHLDCKTEVKPYNTISPDDELTYIIFIDSQDHSVMLNPTPEIFKNVQKMLLHNSGLIWVVPEGASPDAHMIKGLMRTLRLEEAPKNLMVFDEVPLTPVGLKGIVKLAESLRNPEVRRDEDQDFHLHNGTIHLPRMRQLNEVKELFAVEQGIAYRKVQNIWEGGRALEMTIDAAGSPDSIYFRRTNVLQQPMGDDEVLIRVEAAGVSNRDLNLVLGSIPWAPPGYDGAGKVVKTGSHVSHLREGDDIFFLALESSAFATYKKMPAWHVAKIPSGLSITDASTLPLAYTLAVLGLIRTARLRKSDTILIHGAAGAVGQASIAIAQHIGATIFATAGTEAKREFIHQAFGIPKERIFTSRTAAFRDSILSATDNKGVNVIINSLGSEFITETWALAAKFGRFVEISKEAAFQNINLPMRAFDSNVTFSPVDIRELYKHQPDELRDVWSEVVDLLKRDVVKPIKPVTLIPISDFVSALRKLKSGDHLGKIVVTLGKDEKVVAESALSPTEVKLRTDGTYLVTGGTRGIGLDLAYWMIEHGAKNIVLLGRSGATGEEVKKMLKRYEGNDVTIRALACNVGIRDELVNVMEAIKDLPPVRGVVHSALLLSDKLFANSTHEDWQIVNTPRVQGAWNLNELLPADLDFFVLLSSFNGDTGNMGQAIYAGTAGFYDAFSRYRNARGQHTVSIALPIVLDVGYVADNNLTEILKQTLGVVLKMADIRALFKGAVSGPASPFHSNGKATAFKLYMEGQSLQNPPWKYFHPVHTRERLKADKDARLKAGATGGADMFTASWTTAEDPLEGLTEALITKVSAMTMIERDEVLPDAPLTSYSLDSLVSVELRNWIRRETTVELPLSSITQAESLRALATDILSQRVI

**Ⅵ Preu6**

MSNSTRDYPISAAFFCPQSRAPPAEYLHALYSFLSQNTLGKAFLRHIASLDEVWPIFSEARDDILRLPDARQNINVLVDWAKGGSSTPIAEARSGVIALPSVFIVQLGQYFRYLEANRLSHGDFIGQLKDIGGVHGYCGGAAAALSVACAADETQLIDHAAVLLRLFVGIGCCIEAVDDWTTTESTVIACRLKYEGQGDELCSRFPEPKSISITGNARTLSELFDYAVGLGLPTHKMEITGKAHNPENAELAKDFINLYRRTPALQLPPTFKLQATVRSNRTAEKLTNEGIIEDMITMIIASQCDWNTLLTRVAEDMKVSGRPFHKMVSFGMNDCVPVTPFNRQRLKTTKFEAHVLIEPLKPSRISAAQYPTFSDDAIAITGASLRLPGANNLDELWDLISKGTDCHREIPKDRFDPHNIYRTSQSGFSKAQKYFGNFLEDIKGFDRAYFSMGVREAANIDPQQRLLLELAVEALEASGYLANHVREAGDPVGCFVGASFIEYLENTGAHPPTAYTAPGTIRAFLCGRLSYYFGWTAPAEVIDTACSASMVAINRAVKSIQAGECEMALAGGVNLITGMNNYLDLAKAGFLSPTGQCKPFDQSGDGYCRSDGAGFVVLKKLSQALVNGDPIMGVIPSIETNQGGLSGSLTVPSSTALQALYKRVLSKSGLEPAQITYVEAHGTGTQAGDPIEVESVRAVLGDPTRAHSLSLGSVKGNIGHCETGAGVAGLLKVLAMIKHGGIPPLASHKALNPKIPALETHHMEIAKQLKPWDVPLRAAFVNSYGAAGSNAAVICVEPPPVVTDGSSLIGTEPQKVTLPVIVSGATRKSLVLNARALASYLSQDGSHLSIHDVAFTVNQRRKRNRFCAEVSGTDLPSLVQSLRAVDSPSFESPGKSKPVVLVFSGQNTNAVALDRTIYDTYPVFKAYIDACDSEIVKLGFPSIMEAIFQKEPISTAVALQGSIFAMQYACARSWIDAGLKPRAIIGHSFGELTALAVSGALSLADSLKLVTCRGHLIDTKWGEERGGMLVIHADVATVERFQSRFKAQHDGAELEIACYNSPTTTVVAGPVAYMDAAEQMLATDPDFQGLRKLRIVTSNAFHSSLSDPILADLDSMADTLTWNEPSIPLEACTSEGLASIKEWSASRHTRGSVYFTKAVERIEGRLGACIWVEAGLDSAIIAMARKASSKPDSQVFQSVSTKAGATSFIDGIVNNLWRQGVPLSHLNALSATVKPNPVWLPPYQFEREQHWTEHIDRATEASQASTTSDTIQSTPTQTVQSPPKLISRLASLQYQINTQCERFQKITEGHAVLYEPLCPASLYMECVVMALQELAGDLGSRTLDFENLDFHAGLGLQTDRRVLLDLEEARPHSWTFKVQSTKAGSSRSLLHCSGRVILTESSVPTTFQRLVDGPRSRLDQDKDAEKLMSSRAYGLFSNIMTYSEFLKPISSIILRENESLATIKLPPNQPGLHESTAWKRCDAVFLDGFISSSGLLLNSSSVVQSGHVLIAVGVERAILTAAFQASLASSWQAYATFTMVGETHALCDVFACTPDGEVVAMMTGVRFNKMEISKLAKSLSSVNASSPTGGRTQPPAAPKTQAQPMASRPSPTPLQVSFATAEPAAPEPVQQSTAALARNDIGPVLKSLISNYTGLIEEDVSEDSPLVDLGLDSLSSVEFASEIGTKFGVTLDADTVGDLTLHSLCQRLSGTSNVVSQKMSETPAAAPVKELIETVPSPIVTFSSPVSNSITSVLKSLLGSYTGLQEEDMPDDVPLIDLGLDSLSSVEFASELNDKMGADIDSAVVADMTLSALEQQLGASATPPSTTGSSTPGDISTAATTPYATGASTPDYLVHGNKPSISNGVVAAKDSYQVKTVEYKRVSGVPIHADIYVPLVQRVSPMPLALMIHGGGHMTLSRKAVRPTQAKYLLSHGFLPISIDYRLCPEVNLIDGPIADVRDAYVWACQNLGTHLAEHSISVDGGRVVVVGWSTGGHLAMSLGWSLEEAGVPPPKAVLSFYAPVDFESGELDNQKNPALPKPRMTLDQITKALPRTPVTQYGASSTDETNLGWLHPGDPRSELLLHVFHSDIGLPLILHGLPISGSGRPSPSLVASISPLARLRNGSYTIPTFIIHGTKDVIAPYAAAERFVKIMSEKGVKSGFLSLSGTGHVFDVTMKPDSKGWEDKVKPGLDFLIQNA

**Ⅶ Preu7**

MAWIGTHNPELLSFTPSGTSDMSIEQVNAGEIRVAFFSNEFPHDDLGNLFRQLHIHSNDRRHLLLARFIHEATSALHEEVRLLPNALRALIPPFETIFNLVDHEALRNGRLGGAINGALLCAVQLATLIGFYEDQPNGHSEDTDSVETLLAGLGIGLLSTAAVSLAPTLTHMPIAGAEVVRIAFRLGILVDRVSEHLQPRPPHDDGHGDSWAYVIPDVTAEEVQRELDEIHASEATPTASKLFLSAISQTSVTISGPPARLKHIFLVSDFFRSQRFVSLPVYAGLCHAKHIYNKDHVDSVIDTPSLEALDAASAPRIPIYSTNTGKPFPAKTSKELFHLIVEEILTQAIQWDKVVEGILTRARDVQASGCQILVFRNSLPVHDLMAGFSGLDQIKADTLELIPWISKPDQCPTVPSGTATSKIAIVGMSCRMPGGATDTEKFWQILEQGLDVHRKIPADRFDVDSHYDPEGKRLNASHTPFGCFIDEPGLFDASFFNMSPREALQTDPMQRLALVTAYEALERSGFVANRTAASNAHRVGTFYGQASDDYREVNTAQEISTYFITGGCRAFGPGRINYFFKFSGPSYSIDTACSSGLATIQIACTSLWAGDTDTAVAGGMNVLTNSDAFAGLSQGHFLTKTPNACKTWDAEADGYCRADGIVSIVMKRLEDAERDNDNIIGVILGAGTNHSADAISITHPHAGAQSYLTRQVLSQAGVDPLDVSFVEMHGTGTQAGDAQEIISVTDVFAPTKRRRTAKQPLYIGSVKANVGHGEAVAGPTALLKVLLMLQKEAIPPHVGIKNTINPGFPKDLEKRNVNIPYQKQPWPRTPGKKRIAVVNNFSAAGGNSTVLIEEPPAREHSDVVDPRSTHVVVISAKSKVSLKGNLQRMIAYLEANPDVSLPDLAYTTTARRHHHNHRVAVATSGVAHLKKQMGSYLPSVETHRPIPSTGAPPVVFTFTGQGASYRSMNLELFHHCSYFRTELLELDSLAQGQGLPSFIQALDGSHEQDYAHSPTVTQLALVCTEIALAKYWESLGVRPDAVVGHSLGEYAAMHVAGVLSANDTIFLVAQRAMMLEKNCKTGTHKMVAVRASLDEIDSVAEIASLAESRGVAGVRPFEVACINGPRDTVLSGLSKEMDEVSDILTRAGFKCISLDVAFAFHSAQTDPILDEFEAIATTGALFQPPTLPIISPLLGKVIFDERTVNANYLRRATRETVNFLAAIESAQKTSTIDETMVWIEIGPHPVCLGFAKSILPTVNAAVPSLRRGENNWQTMSQSLGTVFSSGIEVRWDEFHRSFEKHLRLLNLPTYAWNDKNYWIQYNGDWALTKGNTFYDAEKTSKAPSRVPVSAKHSSLRTSTVQHIIEETFSATACTVVMQSDLMQPDFLAAAHGHKMNGCGVVTSSIHADIAFTLGEYLMKKLKPKAKDISMNIADLVVTKGLVANKNTESPQFIQVSATTHDIEAGVVHLEWRNVVSGEALAENFATAKLHYESSGDWLKTWIPITHFVRSRIEALEGLAADGFANRLTHDMAYRLFANNLVDYAQKYRGMQSVVMYGLEAFADVALTAEKGGVWTVPPYFIDSVAHLAGFVMNVSDANDTENNFCVTPGWRSMRFAKPLIPGGRYRSYVKMIPTPEDPTVFLGDVYILQESVVIGMVGAIEFRQYPRILLNRFFSAPDDSSHLHSSTSVAKPKQTSSTTTSQAKSVPVTVPELVTATVIPTEPTPVEAAAPAATKGVTVEVKVDSDSTAAKAMALVAAEAGLELADLEDDVSFGELGIDSLMSLVIAEKFREQLGVVVNGSLFLEYPTMGDLRAWLVEYYS

**Ⅷ Preu8**

MNVLVLGDQVADHLQLLENTYHRKNNASVKGFLDRATDVLLSEIAKLPKRQRENIPEFRTILNLVQLYRERGLAIPHVESALVTVSQLAYYIGYFADYPTDLPNPENKRVLGLCTGQLAASVVASSHTLDELLPIALEAVKLAFRTGLEVACQGEAIEQGPLSSEKWSTVVQDITEDEAMSLIDNFHKERKIPVSAHAYISAVAPTSITVSGPPSTTKELFEIEELSKKRKTPIPVFAPYHASHLYDASVIDRIIGDSAEVLKRYRSRALFHSASTGKCHITTDTLELVRVALREILLEPVRWSSLIEECVSQVLESGDTECTVFPIGATMVTNSLVSTLKAAGQSSLSVRVSKPWGPDWEGTKGRTQNDKIAIVGMSGRFPSAASHEELWELLAKGLDVHREIPSDRFDAQAHCDPSGKGKNKSHTPYGCFIDEPGMFDPRFFNMSPREAAQTDPMGRLALTTAYEALEMSGYVPNRTPSSMLNRIGTFYGQTSDDWREINAAENVDTYFITGGVRAFAPGRINYYFKFSGPSFSIDTACSSSLAAIQLACTSLWAGDCDTACAGGLNVLTNPDIFSGLSKGQFLSKTGGCKTYDNDADGYCRGDGCGTVILKRYEDAIADKDNILGCILGAATNHSAEAVSITHPHAGAQEFLYKKVLANAGVDAHEITYVEMHGTGTQAGDGIEMTSVTNAFAPRNRQRRPDQPLYLGAIKANIGHGEAASGINSLVKCMMMLKKNAIPANVGIKGVMNKTFPKDLAQRNVHIETEMVPYPRKGAEKRKMFLNNFSAAGGNTAIILEDGPLREAPKGVDPRTSQVVTITGRSISSLKKNIDNLIKYLDQNPDTTLPSLAYTTTARRIQHNYRVAVVVSDISQVKDALKNQIKDSYSPVAMVSTKTAFTFTGQGSQYTGLGKKLYEEMGSFKSDIHQLDNLARLHSLPSIIPLLDGSTDVAKLSPVVVQLGMACIQVALSRMWASWGVTPSAVIGHSLGEYAALHVAGVISASDMVLLVGRRAQLLEKECTAHTHGMLAVKGGVESISDALGDKMIEIACMNGPEETVLCGKVDVIESTADALATKGFKATKLNVPFAFHSAQVEPILEKFQDVATSVKFKKPVVPVLSPLNGEVIREAGIIGPKYLADHARRTVDFWHALSAGKDEKAFDEKTAWLEIGAHPVCSGMVKSSLGGSPCTAGSLRRNEDPWKTLANSVSTLFLAGVGIDFPEYHRQFDDAHELLTLPTYAFDNKKYWLDYHNNWTLTKGEARTDAAPKTIEAPAEVKSKLSTTSCQRIVREELHANSGTVVVQSDLSDPKLRATISGHQVNGTPLCPSSLYADQAMTLADYLYKQLRPNLPTPGLNVCAMEVPKTLIPQYPPPAGGQHLQIEATADLEHNRVEVRFRTVPADGSKILAEHAFGTVKYEDVSQWKEEWARTQYMVQTQIDLLKQKLVSGSAHKVLRGMAYKLFKALVSYADNYRGMEEVILDGKQTEATATVKFQTTPEDGSFFCSPYWIDSLAHLSGFIVNASDHLDSENSVYISHGWGSIKISKQLSPEKRYQSYVRMQPAPGNISVGDVYILEGEEVIGLVTGLKFQNIARRVLNIMMPPAGGAAKAAGGKAAPAKKAASPTLAPAKAAKPAAKTSKPSKARAKPAADSTTSRVMKIIATETDVDMAELVDEAAFENLGVDSLMSLTISAKFREELDLEISSTLFTDHPTVGQMKKFFSQYDGAPIPDDGDDSDGTDEPSNFSTPSYGADNASTPPSSAPSVNGKSSPENHEVLESTEVSLARKIVAEEMGVDVAEITDKADLSEMGMDSLMSLTILGALRESTGIDLPSTFLVTNVTIEDIENELGMRPKPKPKAEAAPPKSSAKASPSANKQPQLSAVNEKLKNIVDVSQYPPANSVLLQGNPKIATKKMFLVPDGSGSATSYISVPPISPDLAVFGLNCPFMKSPEKWTCGVEGVSALYLAEIKRRQPKGPYIIGGWSAGGVMAYEVTQQLVNSGEVVERLVLIDAPCPVALDPLPARLHIFFDQIGLLGTGKPGGTPKWLLPHFASAIQNLKDYEPIPMDPQRAPEVFAIWCTDGVCPNPDDPRPPPGEGEDPAPMKWLLNNRTDFEDNGWAQLLPKKNFTYAVMGGNHFTMMKGDHGAKLGEHLKEGLKL

**Ⅸ Preu9**

MYALHLAVNAIRAGDCESAIVASANWIGDPGVQIALDKLGALSASARCHTFDLRAEGYARGEGFGAIYLKKTSLAISSGSPIRAMIRGTAMNSNGRTGGITRPSASGQEAVIREAYRNAGRLSFKDTGYFECHGTGTYVGDPIEVAAVGRVFASDRNDSAPLLVGSVKSNVGHSEGASALAAVMKVVLALENGAIPPIYDLQTRNPNIDFEGARVLPVTEVTEWPKDRLRRASINSFGYGGANAHCIIDHVNNVLADYEAPGVYRSIDDSSRNGVQNGHLNEFAANGTTNAPSRDHRNGITDGRADGNTNGHPNANGDVGGNPINGQANGDALMIHHPPMVRIPKKMRNAITRRLVLLPLSAHNETSLDRNWAEISQVLPTFPLFDIAYTLGARRSRYPQRTFTVVNSHTPVQTQSLVLDRKPTRAPLQTATIGFVFTGQGAQWHAMGADLWDYAVFRAVIQYQDNVLACLQNAPTWSLESVLRGDCEAGLVQTAAVSQAACTAVQVGLVDLLASWSIRPAGVVGHSSGEIAAAYASGRISAAEAIVAAYLRGQAVSLNGREGAMLAVGLGPEQVAEYVQEREAEVKVAAINSPGSVTLSGDVSAIDQLAATLTAEGIFNRKLHTGGNAYHSHHMMAIGNAYMAMLRDADGHMHAHRGDRYPHVSWVSSVTPTKSTPTSSTDGTDVVDLGPYWRSNLESRVRFAEAISRLVESIPVSVLVEIGPHPALKSPVEQILKSVGKTAGYVGTLKRNEDGQQSLLQLAGTLFTLNAVVDLAAVNAVDTADGSGSECGATCTSLPRYQYTYGGLNYHESRHSKEYRHRMELRHDLLGSKVVGTARLRPQWRNILRIKDVPWLGHHRLVPDAILPGAAYMAMAVEAVGHIYRGGVEAHVTVTGFELSDVTIDRSLVVPEDDYGVEVLTSLELTFDFFDVLTTATFSISSVGRDTGEWVQHCTGCVKLIIKSSNVDDISHTIQVPETLRPVDVRAWYTPTGRFQKVGLGYGPAFQPLTDVSSDGNHLAVASVALHTPSEHGAVKGGESDYPLHPAALDGAIQLGLIACHGGRPSEVTAAFVPVHLSRMYLSNDINDATAYGDAPTVVACGERRGIRSAHLDIDMRSPNGKVLLRVERLRCVSYSRISSDSTDRAFSSPFTRLVWRPDIRTISNAQARHRYPAPQGKQSSAWAVTNKLGHFVVQSIYETFGKLADGNRPHPSGDVGHFFAWIQKKGQHDQSPSMLEARKLACENRLLESIDELVKQAFHVLEVQIAKLLHDKMSDILFERRTGIDVIIGEGLLTPLYQSGLLMTGIYPQLHRIISGLAHADPNARILEIGGGTGGATRIAMNALNGPNGIKAYRDYTFTDISAGFLSGARELLGHLPDMKFSVFDIERDPVEQGYDEQTYNLIIACQVLHATSNMHRTLTNCRRLLKPGGRLVLLETNENFIVPGVVVGTFTGYWAGIPDGRVDAPFQSLDSWDRSLRAAGFSGLDVVLDDFPEPQNTTSVMLSTVPIHIPEKDVSGTLVHVLHSTPEVPRLVPKVVEGFEERGITATISSLGNGPVQLPPASHAVIFYDEQDLLANSSEKSLGVFQHLSENSATLLVLTSCGTVNGLNPDGALIPGLLRVLRNENPATEYGSIDIDATHFNVDSSEEQEIARRIVDCELDLRRSVLPEELESTPPDREFVWQKGCMWVSRHVPDAGFHSEHGLDNKSMKPELLPLSSQGAVRAVFESPGVPNSLCFASYEEMKEPLQPDYIDVEVAAIGLNSQDIDHWTGRVNANHLSSEYAGVVTAVGTNVYNLKVGDRVYGLGKGQFGNWTRGPSVLAQKLQPEDKMIQMASMPLAYTTATYVLEQIARLRKGQSVLIQSGAKDIGLAILNLAKTKEAVVFAIVETPEQVDFLTAKMGMPASRIISTIPTLAVLRRAAQGTCNGKFDVIVSTVSGEAQQSFPSMLSHLGHWIDMSQNEPQTLSTVNGRLLLHNASYCFVDPTAIFDTSPVLAAEIKQTVDKHYRKGLIGPIPRIEESDVSQIGSSLGNLANMIGKLVVSFENPESLVRMVPSPPSVRFDPKSFYVITGVLGGLGQSLVQWMASRGARHLALLSRRHVSSVPEAEKFITSLSNRGINVSCLVCDVSDAAQVNKVIKDLSSH

# References

Braesel, J., Fricke, J., Schwenk, D., and Hoffmeister, D. (2017) Biochemical and genetic basis of orsellinic acid biosynthesis and prenylation in a s*tereaceous basidiomycete*. *Fungal Genet. Biol.* 98, 12-19. doi:10.1016/j.fgb.2016.11.007

Cai, Y., Rao, L., and Zou, Y. (2021) Genome mining discovery of a C_4_-alkylated dihydroisocoumarin pathway in fungi. *Org. Lett.* *23*(6), 2337-2341. doi:10.1021/acs.orglett.1c00458

Chung, K. R., and Liao, H. L. (2008) Determination of a transcriptional regulator-like gene involved in biosynthesis of elsinochrome phytotoxin by the citrus scab fungus, *Elsinoë fawcettii*. *Microbiology (Reading)*. 154(Pt 11), 3556-3566. doi:10.1099/mic.0.2008/019414-0

Cochrane, R. V., Gao, Z., Lambkin, G. R., Xu, W., Winter, J. M., Marcus, S. L., et al. (2015) Comparison of 10,11-dehydrocurvularin polyketide synthases from *Alternaria cinerariae* and *Aspergillus terreus* highlights key structural motifs. *Chembiochem.* 16(17), 2479-2483. doi:10.1002/cbic.201500428

Coppin, E., and Silar, P. (2007) Identification of PaPKS1, a polyketide synthase involved in melanin formation and its use as a genetic tool in *Podospora anserina*. *Mycol. Res.* 111(Pt 8), 901-908. [doi:10.1016/j.mycres.2007.05.011](https://doi.org/10.1016/j.mycres.2007.05.011)

Ehrlich, K. C., Chang, P. K., Yu, J., and Cotty, P. J. (2004) Aflatoxin biosynthesis cluster gene *cypA* is required for G aflatoxin formation. *Appl. Environ. Microbiol.* 70(11), 6518-6524. doi:10.1128/AEM.70.11.6518-6524.2004

Ehrlich, K. C., Li, P., Scharfenstein, L., and Chang, P. K. (2010) HypC, the anthrone oxidase involved in aflatoxin biosynthesis. *Appl. Environ. Microbiol.* 76(10), 3374-3377. [doi:10.1128/AEM.02495-09](https://doi.org/10.1128/AEM.02495-09)

Fedorova, N. D., Khaldi, N., Joardar, V. S., Maiti, R., Amedeo, P., Anderson, M. J., et al. (2008) Genomic islands in the pathogenic filamentous fungus *Aspergillus fumigatus*. *PLoS Genet. 4*(4), e1000046. doi:10.1371/journal.pgen.1000046

Gressler, M., Hortschansky, P., Geib, E., and Brock, M. (2015) A new high-performance heterologous fungal expression system based on regulatory elements from the *Aspergillus* *terreus* terrein gene cluster. *Front. Microbiol*. 6, 184. doi:10.3389/fmicb.2015.00184

Itoh, T., Tokunaga, K., Radhakrishnan, E. K., Fujii, I., Abe, I., Ebizuka, Y., et al. (2012) Identification of a key prenyltransferase involved in biosynthesis of the most abundant fungal meroterpenoids derived from 3,5-dimethylorsellinic acid. *Chembiochem.* 13(8), 1132-1135. doi:10.1002/cbic.201200124

Kim, J. E., Han, K. H., Jin, J., Kim, H., Kim, J. C., Yun, S. H., et al. (2005) Putative polyketide synthase and laccase genes for biosynthesis of aurofusarin in *Gibberella zeae*. *Appl. Environ. Microbiol.* 71(4), 1701-1708. doi:10.1128/AEM.71.4.1701-1708.2005

Kim, Y. T., Lee, Y. R., Jin, J., Han, K. H., Kim, H., Kim, J. C., et al. (2005) Two different polyketide synthase genes are required for synthesis of zearalenone in *Gibberella zeae*. *Mol. Microbiol.* 58(4), 1102-1113. doi:10.1111/j.1365-2958.2005.04884.x

Korman, T. P., Crawford, J. M., Labonte, J. W., Newman, A. G., Wong, J., Townsend, C. A., et al. (2010) Structure and function of an iterative polyketide synthase thioesterase domain catalyzing Claisen cyclization in aflatoxin biosynthesis. *Proc. Natl. Acad. Sci. U S A*. 107(14), 6246-6251. doi:10.1073/pnas.0913531107

Lackner, G., Bohnert, M., Wick, J., and Hoffmeister, D. (2013) Assembly of melleolide antibiotics involves a polyketide synthase with cross-coupling activity. *Chem. Biol.* 20(9), 1101-1106. doi:10.1016/j.chembiol.2013.07.009

Lünne, F., Niehaus, E. M., Lipinski, S., Kunigkeit, J., Kalinina, S. A., and Humpf, H. U. (2020) Identification of the polyketide synthase PKS7 responsible for the production of lecanoric acid and ethyl lecanorate in *Claviceps purpurea*. *Fungal Genet. Biol*. 145, 103481. doi:10.1016/j.fgb.2020.103481

Malz, S., Grell, M. N., Thrane, C., Maier, F. J., Rosager, P., Felk, A., et al. (2005) Identification of a gene cluster responsible for the biosynthesis of aurofusarin in the *Fusarium graminearum* species complex. *Fungal Genet. Biol.* 42(5), 420-433. doi:10.1016/j.fgb.2005.01.010

Matsuda, Y., Awakawa, T., and Abe, I. (2013) Reconstituted biosynthesis of fungal meroterpenoid andrastin A. *Tetrahedron.* 69(38), 8199-8204. doi:10.1016/j.tet.2013.07.029

Matsuda, Y., Iwabuchi, T., Fujimoto, T., Awakawa, T., Nakashima, Y., Mori, T., et al. (2016) Discovery of key dioxygenases that diverged the paraherquonin and acetoxydehydroaustin pathways in *Penicillium brasilianum*. *J. Am. Chem. Soc.* 138(38), 12671-12677. doi:10.1021/jacs.6b08424

Matsuda, Y., Wakimoto, T., Mori, T., Awakawa, T., and Abe, I. (2014). Complete biosynthetic pathway of anditomin: nature's sophisticated synthetic route to a complex fungal meroterpenoid. *J. Am. Chem. Soc.* 136(43), 15326-15336. doi:10.1021/ja508127q

Mayorga, M. E., and Timberlake, W. E. (1992) The developmentally regulated *Aspergillus nidulans* *wA* gene encodes a polypeptide homologous to polyketide and fatty acid synthases. *Mol. Gen. Genet.* 235(2-3), 205-212. doi:10.1007/BF00279362

Perez-Cuesta, U., Aparicio-Fernandez, L., Guruceaga, X., Martin-Souto, L., Abad-Diaz-de-Cerio, A., Antoran, A., et al. (2020) Melanin and pyomelanin in *Aspergillus fumigatus*: from its genetics to host interaction. *Int. Microbiol.* 23(1), 55–63. doi:10.1007/s10123-019-00078-0

Regueira, T. B., Kildegaard, K. R., Hansen, B. G., Mortensen, U. H., Hertweck, C., and Nielsen, J. (2011) Molecular basis for mycophenolic acid biosynthesis in *Penicillium brevicompactum*. *Appl. Environ. Microbiol.* 77(9), 3035-3043. doi:10.1128/AEM.03015-10

Sanchez, J. F., Entwistle, R., Corcoran, D., Oakley, B. R., and Wang, C. C. (2012) Identification and molecular genetic analysis of the cichorine gene cluster in *Aspergillus nidulans*. *Med. Chem. Commun*. 3(8), 997-1002. doi:10.1039/C2MD20055D

Tominaga, M., Lee, Y. H., Hayashi, R., Suzuki, Y., Yamada, O., Sakamoto, K., et al. (2006) Molecular analysis of an inactive aflatoxin biosynthesis gene cluster in *Aspergillus oryzae* RIB strains. *Appl. Environ. Microbiol.* 72(1), 484-490. doi:10.1128/AEM.72.1.484-490.2006

Tsunematsu, Y., Ichinoseki, S., Nakazawa, T., Ishikawa, N., Noguchi, H., Hotta, K., et al. (2012) Overexpressing transcriptional regulator in *Chaetomium globosum* activates a silent biosynthetic pathway: evaluation of shanorellin biosynthesis. *J. Antibiot (Tokyo).* 65(7), 377-380. doi:10.1038/ja.2012.34

Wang, S., Xu, Y., Maine, E. A., Wijeratne, E. M., Espinosa-Artiles, P., Gunatilaka, A. A., et al. (2008) Functional characterization of the biosynthesis of radicicol, an Hsp90 inhibitor resorcylic acid lactone from *Chaetomium chiversii*. *Chem. Biol*. 15(12), 1328-1338. doi:10.1016/j.chembiol.2008.10.006

Wheeler, M. H., Abramczyk, D., Puckhaber, L. S., Naruse, M., Ebizuka, Y., Fujii, I., et al. (2008) New biosynthetic step in the melanin pathway of *Wangiella* (*Exophiala*) *dermatitidis*: evidence for 2-acetyl-1,3,6,8-Tetrahydroxynaphthalene as a novel precursor. *Eukaryot. cell*. 7(10), 1699-1711. doi: 10.1128/EC.00179-08

Winssinger, N., Fontaine, J. G., and Barluenga, S. (2009) Hsp90 inhibition with resorcyclic acid lactones (RALs). *Curr. Top. Med. Chem.* 9(15), 1419-1435. doi:10.2174/156802609789895665

Xu, Y., Espinosa-Artiles, P., Schubert, V., Xu, Y. M., Zhang, W., Lin, M., et al. (2013) Characterization of the biosynthetic genes for 10,11-dehydrocurvularin, a heat shock response-modulating anticancer fungal polyketide from *Aspergillus terreus*. *Appl. Environ. Microbiol*. 79(6), 2038-2047. doi:10.1128/AEM.03334-12

Yeh, H. H., Chang, S. L., Chiang, Y. M., Bruno, K. S., Oakley, B. R., Wu, T. K., et al. (2013) Engineering fungal nonreducing polyketide synthase by heterologous expression and domain swapping. *Org. Lett.* 15(4), 756-759. [doi:10.1021/ol303328t](https://doi.org/10.1021/ol303328t)

Yu, J., Bhatnagar, D., and Cleveland, T. E. (2004) Completed sequence of aflatoxin pathway gene cluster in *Aspergillus parasiticus*. *FEBS Lett*. 564(1-2), 126-130. doi:10.1016/S0014-5793(04)00327-8

Yu, J. H., and Leonard, T. J. (1995) Sterigmatocystin biosynthesis in *Aspergillus nidulans* requires a novel type I polyketide synthase. *J. Bacteriol.* 177(16), 4792-4800. doi:10.1128/jb.177.16.4792-4800.1995

Zhang, S., Schwelm, A., Jin, H., Collins, L. J., and Bradshaw, R. E. (2007) A fragmented aflatoxin-like gene cluster in the forest pathogen *Dothistroma septosporum*. *Fungal Genet. Biol.* 44(12), 1342-1354. doi:10.1016/j.fgb.2007.06.005

Zhou, H., Qiao, K., Gao, Z., Meehan, M. J., Li, J. W., Zhao, X., et al. (2010) Enzymatic synthesis of resorcylic acid lactones by cooperation of fungal iterative polyketide synthases involved in hypothemycin biosynthesis. *J. Am. Chem. Soc.* 132(13), 4530-4531. doi:10.1021/ja100060k
